# Ginger-derived carbon quantum dots as sustainable inhibitor of steel corrosion and barite precipitation

**DOI:** 10.1038/s41598-025-08684-x

**Published:** 2025-07-16

**Authors:** B. A. Abd-El-Nabey, S. El-Housseiny, D. E. Abd-El-Khalek, M. A. Abd-El-Fatah

**Affiliations:** 1 Faculty of Science, Chemistry Department, Alexandria University, Alexandria, Egypt; 2 Faculty of Sciences, Chemistry Department, Jouf University, Sakakah, Saudi Arabia; 3 National Institute of Oceanography and Fisheries (NIOF), Cairo, Egypt; 4 Faculty of Education, Alexandria University, Alexandria, Egypt

**Keywords:** Carbon quantum Dots, Ginger – Corrosion – Inhibition, Barite precipitation- EIS, PDP, Materials science, Nanoscience and technology, Chemistry, Electrochemistry, Physical chemistry, Surface chemistry

## Abstract

A one-step pyrolysis was used to synthesize ginger carbon quantum dots (G-CDs), which were described using XPS, FTIR, XRD, EDX, and TEM to validate the different integrated functions, particle dimension, morphology, component composition, amorphous construction, and bonding types. The dual action of the synthesized G-CDs as corrosion and scale inhibitors was examined. Scale inhibition characteristics of G-CDs for precipitation of BaSO_4_ were investigated using the conductivity technique. Results indicated that G-CDs have an effective inhibition of 84.9% at 200 mg/L. Application of Langmuir and Flory–Huygens isotherms along with the Kinetic-thermodynamic model on the experimental findings demonstrated the suitability and applicability of all of them, yielding a (∆G°_ads_) _precipitation_ ≃ − 26.7 kJ/mole, suggesting that G-CD adsorption on the scale particle surface is cooperative, involving both chemical and physical interactions, with the physical aspect being the dominant mechanism. SEM and XRD studies indicated that the scale particles (BaSO_4_) were deformed when precipitated in the presence of G-CDs due to their adsorption on the particles’ surfaces.

On the other hand, the inhibition characteristics for the steel corrosion in NaCl solution (0.5 M) were examined using EIS and mass loss method techniques that revealed that G-CDs act as high-efficiency eco-friendly corrosion inhibitor giving 98.3% at 400 mg/L. However, the PDP results indicated that the G-CDs act as mixed-type inhibitor. Application of the adsorption isotherms on the experimental corrosion inhibition data revealed that the three models are applicable, giving (∆G°_ads_) _corrosion_ values equal to − 28.2 kJ/mole, which means that the adsorption process of the G-CQDs is cooperative (physical/chemical). XPS, XRD, EDX and TEM analysis of the steel’s surface upon submersion in a NaCl solution (0.5 M) + 400 mg/L G-CDs solution for 1 h revealed that the corrosion products completely disappeared, the carbon dots were closely attached to the surface of the steel, and the Cl⁻ ions were displaced from the steel surface, which confirms that the G-CDs function as a superior ecologically friendly inhibitor for mitigating the steel corrosion. A mechanism of the inhibition of corrosion of steel in a NaCl solution (0.5 M) by G-CDs was reported, which suggests that in the absence of G-CDs in chloride media, the specific adsorption of the chloride ions on the steel surface and catalyse the charge transfer cathodic and anodic corrosion reactions. However, in the presence of G-CDs in the environment, the strong active absorbable carbon dots surface particles displace the Cl^−^ ions from the steel surface, mitigating the catalytic effect causing inhibition of the corrosion process.

## Introduction

Two major issues facing human society in light of the recent explosion in industrial development are controlling calcareous scaling and preventing corrosion^1,2^. Scale deposition, mainly consisting of calcium carbonate (CaCO_3_), barium sulphate (BaSO_4_) and calcium sulphate (CaSO_4_), other mineral salts, poses significant difficulties in numerous industrial processes and domestic water systems worldwide. These deposits accumulate on surfaces such as pipelines, heat exchangers, and plumbing fixtures, reducing flow efficiency, increasing energy consumption, and accelerating equipment deterioration^3,4^. In addition to preserving operational effectiveness, addressing scale development is essential for minimizing maintenance costs and scaling’s environmental effects.

Scale inhibition has emerged as a critical strategy to mitigate the adverse effects of scale deposition. Common scale inhibitors include phosphonates, polyphosphates, polycarboxylates, silicate inhibitors, organic polymers and chelating agents such as EDTA and NTA, each customized to particular applications and water chemistries^5,6^.

Consequently, considerable efforts have been made to prevent metal corrosion or slow its rate. Carbon steel is extensively utilized in various industrial sectors because of its high strength and excellent mechanical properties. This makes it ideal for applications such as chemical processing, marine structures, power plants, transportation, engineered processes, and construction materials^3,7–9^. Despite its advantages, steel remains susceptible to corrosion when subjected to different environmental conditions. Their efficacy varies according to steel composition, application technique, and environmental conditions.

To prevent environmental issues, it is essential to develop naturally based water treatment agents that are environmentally friendly, sustainable, cost-effective, and readily accessible. Recently, plant extracts have been investigated both as scale and corrosion inhibitors^10^. Previous studies proved the antiscaling performance of various natural extracts because of the existence of functional groups with negative charges in their phytochemicals^12,13^. Also, within the corrosion science field, Yang^14^ published a comprehensive review that documented many organic and sustainable steel corrosion inhibitors in acidic environments, while Praveen et al.^15^ focused on plant extract-based inhibitors.

Among these green inhibitors, the low-toxic carbon-based quantum dots (CQDs) have become a focal point of interest because of their incomparable and special properties. (CQDs) are a novel family of nanomaterials based on carbon discovered by Xu et al. in 2004^16^. It is made up of scattered spherical carbon particles with dimensions under 10 nm. It has been discovered that CQDs possess a variety of valuable properties, including strong optical characteristics, low toxicity biocompatibility, good water solubility, and cost-effectiveness^17–20^.In addition to its numerous applications, its role as an inhibitor for both corrosion and scale formation has recently gained prominence^21–34^.

Recently, Lupine carbon quantum dots (LCQDs) was created by synthesizing the hydrothermal method and further described using various surface techniques. The LCQDs demonstrated a maximum effective inhibition of 89.3% in a solution of HCl (1.0-M) with LCQDs (175 mg/L) for steel corrosion, as assessed by the mass loss method and potentiodynamic polarization along with EIS techniques^35^. Also, novel damsissa carbon quantum dots (DCQDs) were applied to the steel to increase the permanganate phosphate conversion coat’s protective effectiveness^36^. The measurements of EIS showed that 97.4% more protection is provided by the coat when DCQDs (400 mg/L) are incorporated into the coating solution. The surface studies XRD, TEM, EDX and XPS of the surface of the steel coated by permanganate phosphate conversion coat without and with the submerging in HCl (1.0-M) for one hour verified that the DCQDs particles are adherent on the steel surface. Moreover, one-step pyrolysis was employed to create fenugreek carbon quantum dots (FCQDs) then subjected to several surface analyses for characterization. The inhibition efficiency of the prepared FCQDs against CaCO_3_ scale deposition was evaluated using conductivity and chronoconductivity techniques, achieving 65% inhibition at a concentration of 30 mg/L FCQDs^37^. FCQDs’ mechanism of inhibiting CaCO_3_ precipitation was investigated using adsorption isotherms, SEM, and XRD.

Ginger (*Zingiber officinale*), a perennial herb native to Southeast Asia, has been revered for centuries not only for its distinctive flavour in cooking applications but also for its diverse pharmacological properties^38–41^. Ginger has several benefits, including being affordable, having a healthy nature, and ease of availability. The underground rhizome of ginger contains a complex array of bioactive compounds separated into two categories: non-volatile and volatile compounds. Phenolic compounds are abundant in the non-volatile constituents, including paradols, shogaols, gingerols, and zingerone, among others, each known for their antibacterial, anti-inflammatory, and antioxidant properties^35^. Fresh ginger typically contains around 1–3% volatile oils, primarily consisting of sesquiterpenes and monoterpenes, which contribute to its aromatic and medicinal properties^42^. Additionally, gingerols, the major pungent constituents, undergo chemical transformations (such as dehydration to shogaols) during drying or cooking processes, altering their biological activity^43^.

In this investigation, we innovatively focused on (i) Synthesized nanoscale carbon materials with specific structural and chemical properties derived from a low-cost and sustainable source, ginger (*Zingiber officinale*), using a controlled pyrolysis method. (ii) Characterize the chemical structure and morphology of the prepared G-CDs using various techniques that include TEM, XRD, EDX, FTIR, and XPS. (iii) Study G-CDs’ scale inhibition performance towards BaSO_4_ precipitation using the conductivity method, SEM, and XRD analysis; (iv) Investigate the impact of G-CDs for steel corrosion protection in a solution of NaCl (0.5 M) through mass loss strategy and EIS along with PDP techniques; and (v) Discuss the mechanism of BaSO_4_ scale inhibition by G-CDs brine solution in addition to the mechanism of the mitigation of steel corrosion in NaCl solution (0.5-M).

The novelty of this work is that the G-CDs has a dual-nature inhibition behavior, i.e., when their particles are present in the environment, they adsorb on the metal surface, causing corrosion inhibition, and/or adsorb on the surface of BaSO_4_ particles, causing precipitation inhibition.

## Experimental

### Materials

Pure analytical grade NaCl, Na_2_SO_4_, and anhydrous BaCl_2_ were sourced from Sigma-Aldrich, and double-distilled water was utilized throughout the experiments. For the scale inhibition study, 0.01 M Na_2_SO_4_ and 0.1 M BaCl_2_ were produced as stock solutions to create the test solutions. Regarding the corrosion investigation, The working electrode (WE) and coupons were constructed from carbon steel, with the material composition in wt% as follows: Fe = 96.86, Mn = 2.50, Si = 0.35, C = 0.21, *P* = 0.04, and S = 0.04. For the blank solution, an aggressive medium consisting of NaCl solution (0.5 M) was utilized. Double-distilled water was employed to prepare several volumetric flasks containing NaCl solution (0.5 M) with varying concentrations of G-CDs (10, 20, 30, 40, 50, 100, 150, 200, 250, 300, 350, and 400 mg/L).

### Green synthesis of G-CDs

Using a muffle furnace, one-step pyrolysis was carried out to produce carbon quantum dots (G-CDs) from dried ginger, which was purchased from a market as a cost-effective and sustainable precursor. Ginger powder (50.0 g) was pyrolysed for 45 min at 300 °C, transforming to a black substance, indicating G-CDs formation. The pyrolysed material was cooled and then sonicated for 30 min in 250 ml of distilled water, resulting in an aqueous suspension of G-CDs. This suspension was then refined by passing it through filter paper with 0.22 μm-sized pores, yielding purified G-CDs^24^.

### G-CDs characterization

Several analytical techniques contributed to the discovery of the structural analysis of G-CDs. Using a Talos F200S microscope, transmission electron microscopy (TEM) was used to assess particle dimensions and morphology. The Tensor 37 instrument by Bruker was used to evaluate Fourier transform infrared spectroscopy (FT-IR). Thermo Fisher Scientific and JEM-2100 were used to conduct XPS and EDX investigations of the bonding state and elemental composition. The interpretation of X-ray diffraction (XRD) was conducted utilizing a Bruker-AXS/D8 ECO device.

### Exploring **BaSO**_**4**_**scale Inhibition using G-CDs**

#### Assessment of BaSO_4_ scale Inhibition through conductivity testing

In line with previous research, a conductivity test was conducted^44^. A solution of 0.5 mL 0.1 M BaCl_2_ was mixed with a specific volume of the G-CDs stock solution, then the mixture was diluted using distilled water until it reached a final volume of 100 mL. During the titration procedure with 0.01 M Na_2_SO_4_, the stirred solution conductivity using a HANNA conductivity meter was measured. Gradually, the titrant was introduced with 0.2 mL. A temperature of 25.0 ± 0.1 °C was used for the measurements. Experiments were conducted in triplicate under identical conditions to assess the consistency and reliability of the experiments.

### Examination of BaSO_4_ crystals

The BaSO_4_ scale morphology and crystal lattices were investigated utilizing a JEOL-5300 scanning electron microscope (SEM). The scale crystals underwent a vacuum sputter coating process to apply a thin layer of gold before being analyzed with the SEM. To further examine the scale, a Bruker D8 Discover was used to assess X-ray powder diffraction analysis (XRD).

### Examining G-CDs’ corrosion Inhibition performance

#### Weight loss experiment

For 24 h at 30 °C, coupons of carbon steel (5.0 × 2.0 × 0.1 cm) were submerged in 100 mL of varying G-CD concentrations with a NaCl solution of 0.5 M. The coupons were removed from the solution every three hours until the 24-hour period was complete. The sheets were removed, rinsed with purified water, ethanol, and acetone to ensure thorough drying, and then weighed again. To assess the reproducibility and precision of the findings, duplicate tests were carried out under identical conditions. Throughout the test, the coupons were weighed using a Sartorius Secura analytical balance.

#### Measurements concerning electrochemistry

PDP and EIS measurements were made using a PARSTAT 2263.02 SN 194 frequency response analyzer potentiostat. Duplicate tests were carried out to ensure the measurement’s accuracy. A cell with four electrodes was used for the measurements. A Teflon-sealed carbon steel rod possessing a 0.2826 cm² specific surface area served as the working electrode. It was polished with sandpapers of varying grades (100 to 1000). It was subsequently washed with purified water, ethanol, and acetone to ensure complete drying. Saturated calomel electrode (SCE) served as the reference electrode, whereas platinum was used as the auxiliary electrode. Noise during measurements was reduced by connecting a platinum electrode to the reference electrode.

Successive experiments, including EIS and PDP were carried out at 30 °C. To guarantee stability and steady-state conditions, the OCP was observed for 500 s. Impedance was investigated using an applied potential signal with an amplitude of 10 mV across a frequency range of 0.1 to 3.0 × 10^4^ Hz. Polarization curves were carried out within a ± 300 mV range at a rate of 20 mV/min. The meticulous methodology employed in this experiment ensures accurate and reliable electrochemical results^37^.

#### Surface morphology examination of carbon steel

The carbon steel surface morphology was investigated using TEM with a Talos F200S microscope. XRD and EDX were employed to examine the resultant layer on the coupons of carbon steel. For every experiment involving surface characterization, 2 × 1 × 0.1 cm carbon steel coupons were used.

#### Measurements of contact angle

Carbon steel coupons, each measuring 5.0 × 2.0 × 0.1 cm, were polished using various emery paper grades. After polishing, the coupons were dried after being washed with distilled water. Following that, the coupons were immersed for 1 h at 30 °C in a NaCl solution (0.5-M), either with or without the addition of 400 mg/L (G-CDs). The coupons were finally removed after the immersion process, cleaned with acetone and double-distilled water, and allowed to air dry for an hour. A Contact Angle Goniometer (L2004A1) was used to measure the contact angles.

## Findings and discussions

### Structural and morphological properties of G-CDs

The synthesized G-CDs were extensively characterized employing a variety of experimental techniques to determine their surface morphology, elemental content, particle dimension, and chemical structure. X-ray photoelectron spectroscopy (XPS), transmission electron microscopy (TEM), energy-dispersive X-ray spectroscopy (EDX), X-ray diffraction (XRD), and Fourier-transform infrared spectroscopy (FTIR) were among the methods used.

**Fig. 1:**
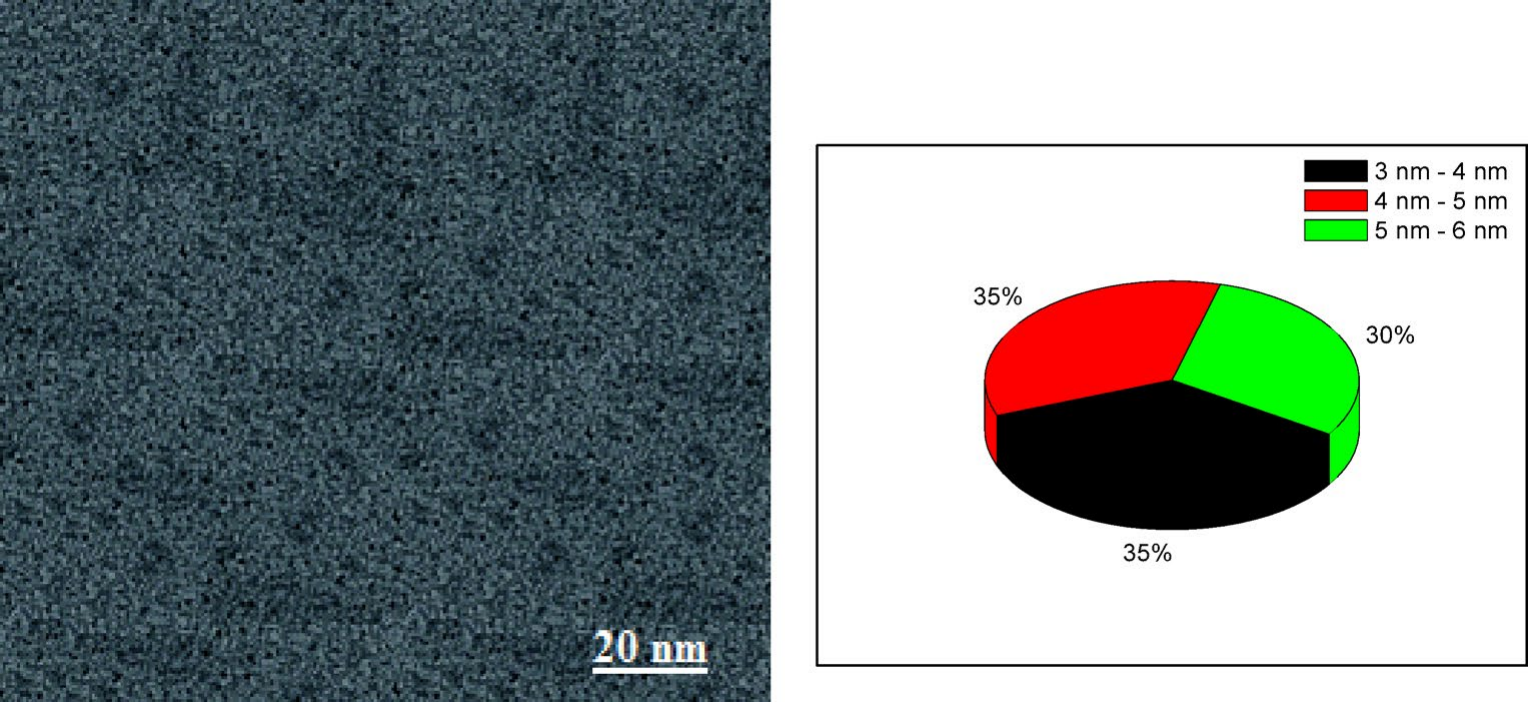
Transmission electron microscopy (TEM) image of G-CDs synthesized from dried ginger, showing size distribution.

The prepared G-CDs TEM image is displayed in Fig. 1. It is evident that they are nearly uniformly shaped nanoparticles, which are typically measured at 3–6 nm. G-CDs are confirmed to be amorphous materials by the diffuse ring visible in the specified region of the electron diffraction pattern^45^. The finding is consistent with the results from X-ray diffraction (XRD).

**Fig. 2:**
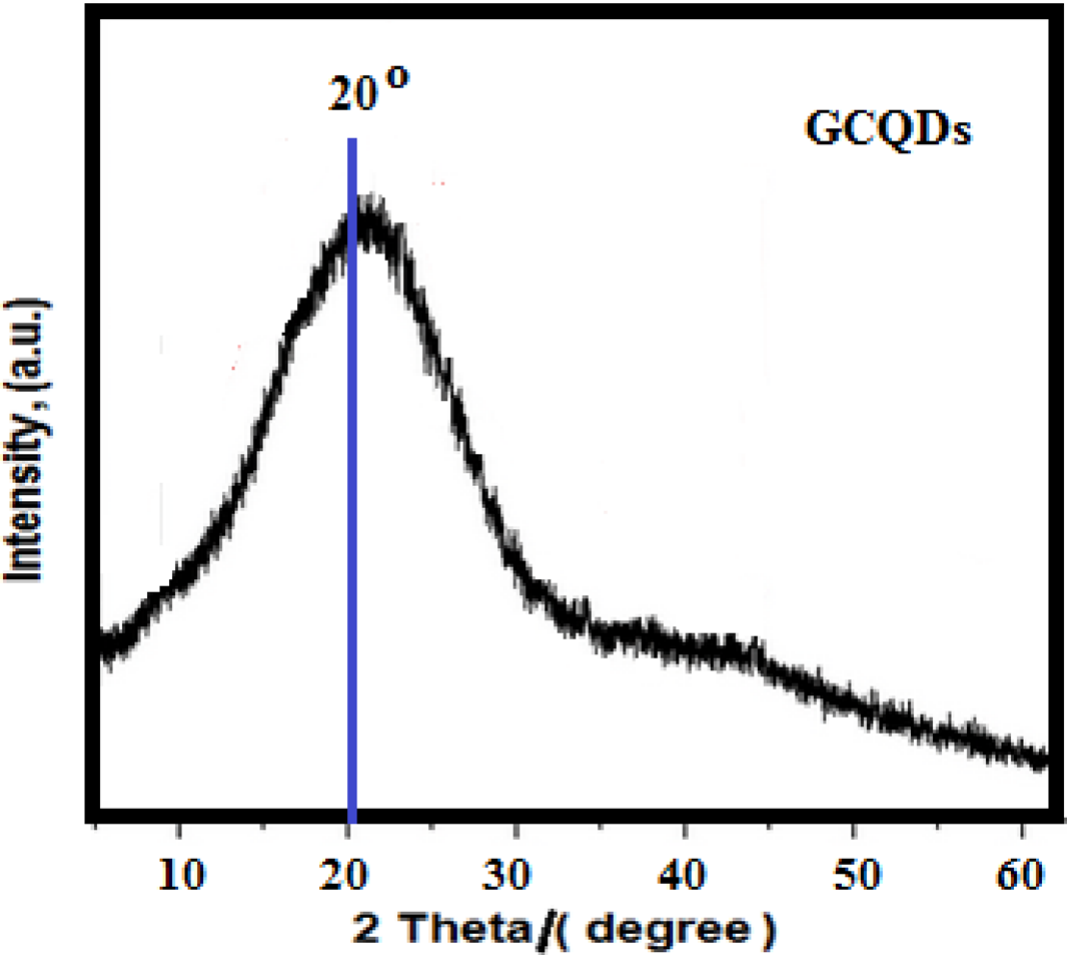
X-ray diffraction (XRD) pattern of synthesized G-CDs.

The amorphous nature of G-CDs appeared as a prominent diffraction peak at 2θ = 20°, as shown in the XRD pattern in Fig. 2^46^. This peak is linked to an increased interplanar distance, which is explained by the existence of hydroxyl and carboxyl as functional groups^47^ or the incorporation of oxygen (O) and nitrogen (N) doped into the structure of G-CDs^48^. The amorphous structure, which is marked by increased surface reactivity, suggests that G-CDs are suitable as agents that hinder steel corrosion in a solution of NaCl (0.5 M) and BaSO_4_ scaling in water.

**Fig. 3:**
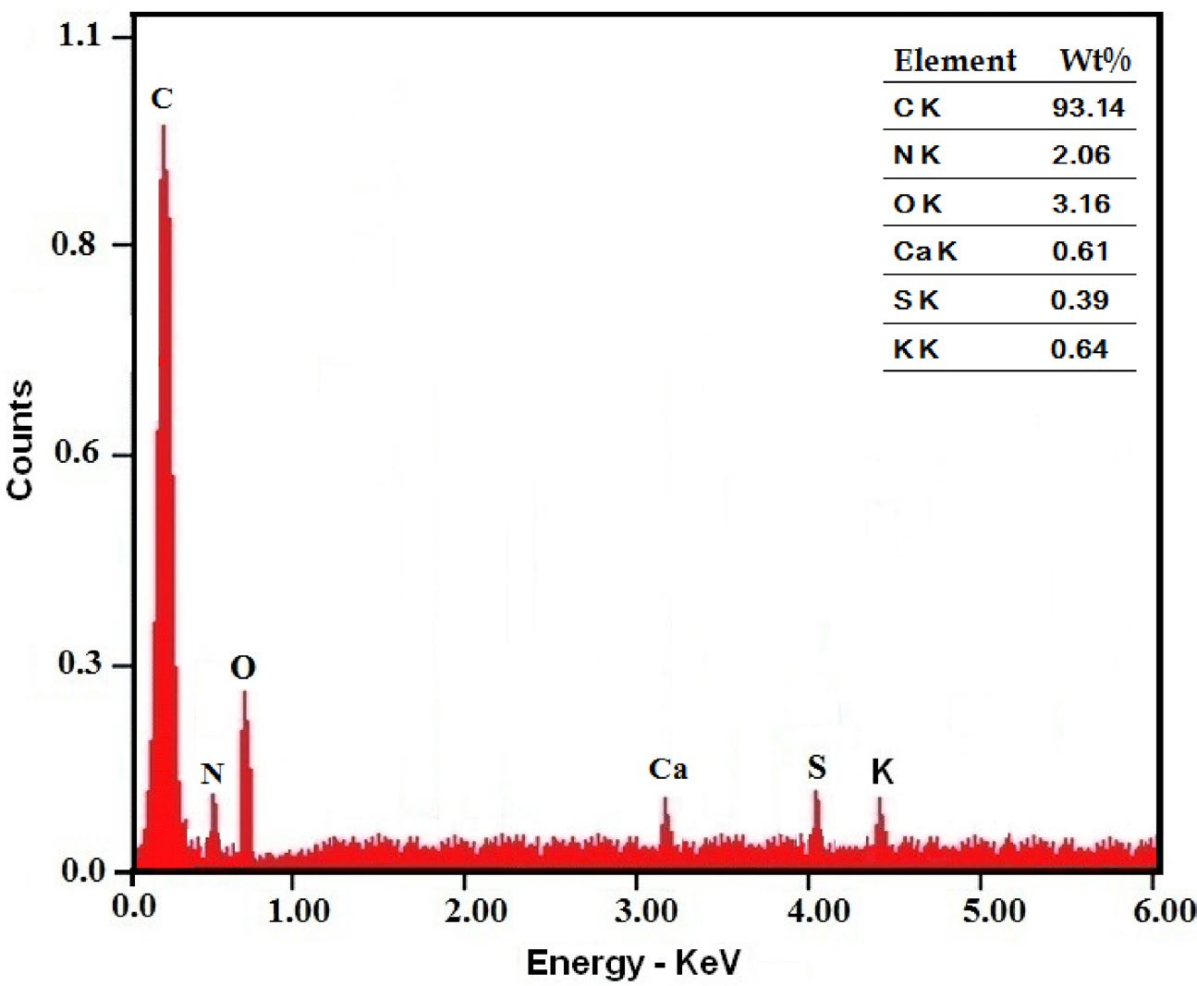
Energy-dispersive X-ray (EDX) spectrum of G-CDs showing elemental composition.

G-CDs elemental composition was analyzed using EDX, as demonstrated in Fig. 3. The findings reveal that the prepared G-CDs are mainly made up of carbon (93.14%), oxygen (3.16%), and nitrogen (2.06%), with trace elements of calcium (Ca), potassium (K), and sulfur (S) also present.

**Fig. 4:**
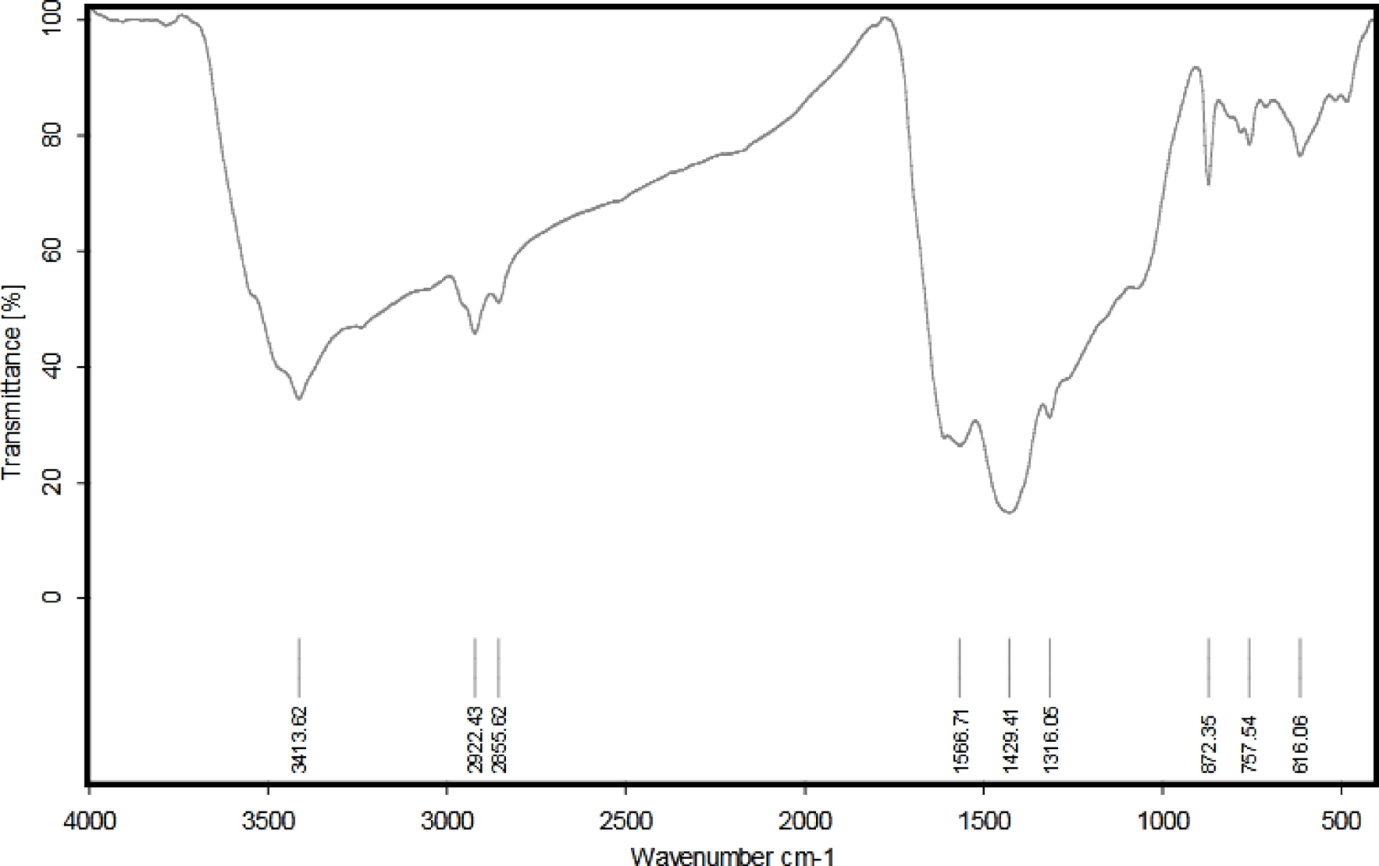
Fourier-transform infrared (FT-IR) spectrum of G-CDs, revealing characteristic functional groups.

The G-CDs chemical composition and functional groups were examined using XPS and FTIR spectra. As seen in Fig. 4, The G-CDs’ FTIR spectrum exhibited several peaks at distinct wavenumbers (3413, 2922, 2855, 1566, 1429, 1316, 872, 757, and 616 cm⁻¹), showing the presence of numerous functional groups. The distinctive absorption peak at 3413 cm⁻¹ is used to relate to the stretching vibrations of the N-H and O-H bonds^49^. The aliphatic C-H bonds (–CH₃/–CH_2_–) exhibit asymmetric stretching and stretching vibrations due to the absorption bands at 2926 and 2855 cm⁻¹^50^. The intense band detected at 1566 cm^− 1^ related to C = C vibrations in the aromatic skeleton. The peak at 1429 cm^− 1^ is caused by the alkanes’ in-plane C–H bending vibrations, although the peak at 1316 cm^− 1^ is preferentially regarded with C–N bending vibrations^51^. The C-C vibration-related band is at 872 cm^− 1^. Peaks at 757 and 616 cm^− 1^ suggest the existence of the wagging vibrations of aromatic C–H, as well as various alkyl halide groups^52^. In conclusion, the FTIR study shows that the G-CDs have a significant amount of unsaturated double bonds in addition to N and O functional groups.

**Fig. 5:**
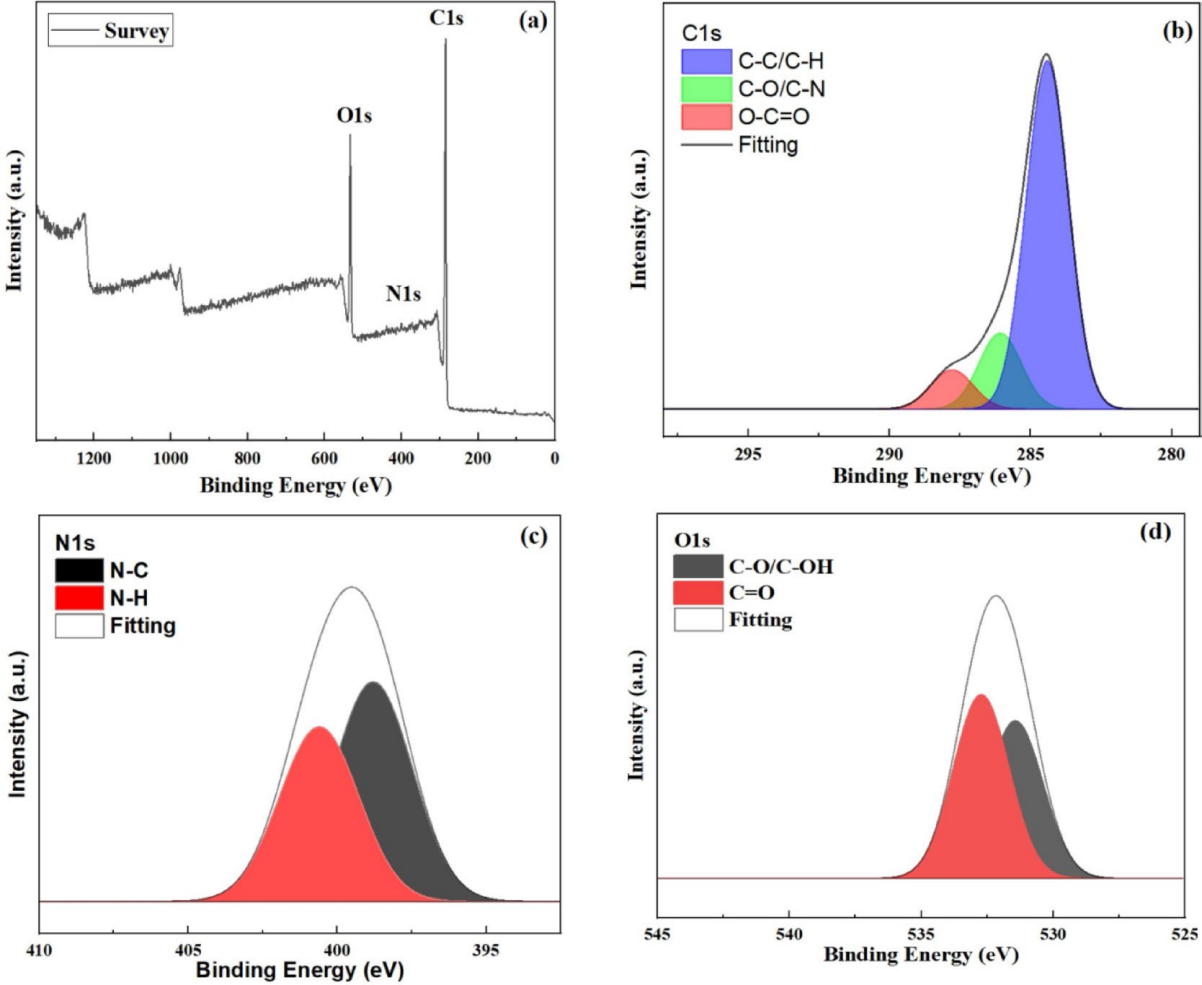
High-resolution XPS spectra of G-CDs: (**a**) full scan; (**b**) deconvoluted C 1s; (**c**) N 1s; (**d**) O 1s.

The complete G-CDs XPS spectrum is displayed in Fig. 5. In Figure 5b, three separate binding energy peaks, identified in the literature as C-C/C-H, C-O/C-N, and O-C = O, are seen in the G-CDs’ high-resolution C1s spectra at 284.3, 286.0, and 288.8eV^53^. The deconvoluted O1s spectra are displayed in Figure 5c. There are two peaks at 533.4 and 531.96 eV that represent the H-C-O and C = O groups, respectively^54^. The two peaks in the N1s spectra, which can be shown in Fig. 5d, are connected to pyrrolic N at 400.4 eV and pyridinic N at 398.8 eV^55^. Previous investigations indicate that there is agreement between FTIR and XPS findings. G-CDs are efficiently doped with oxygen and other heteroatoms, demonstrating the G-CDs’ abundant unsaturated bond and functional group presence. Accordingly, the combined G-CDs can participate in interactions via an adsorption process, which increases their effectiveness in applications involving anti-scaling and corrosion inhibition.

### G-CDs’ anti-scaling performance

#### Impact of G-CDs on suppression of BaSO_4_ precipitation

**Fig. 6:**
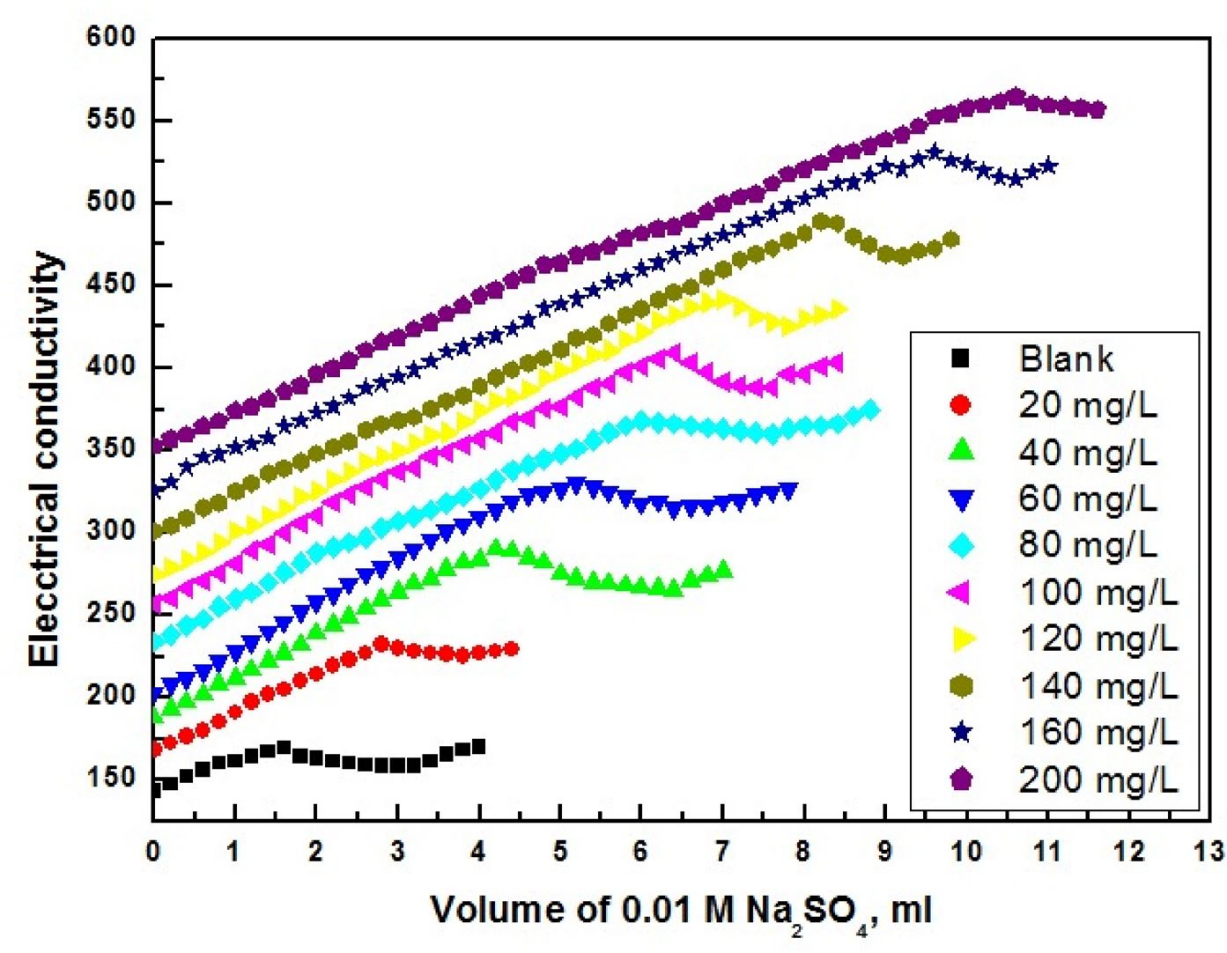
Electrical conductivity changes of a 0.1 M BaCl_2_ solution with 0.01 M Na_2_SO_4_ at 30 °C in the presence of various concentrations of G-CDs.

The effectiveness of G-CDs in inhibiting BaSO_4_ scale precipitation was evaluated through an electrical conductivity test (Fig. 6). During this experiment, BaSO_4_ was precipitated while electrical conductivity was monitored by adding 0.01 M Na_2_SO_4_ solution to 0.1 M BaCl_2_ solution. Clearly, the addition of sodium sulphate obviously increases solution conductivity linearly until it reaches the supersaturating point (S), at which point fast barium sulfate precipitation starts, resulting in a drop in the solution conductivity. Consequently, the addition of G-CDs delays the deposition of barium sulfate scale. Furthermore, with an increase in G-CD concentration, the super saturation point value rises.

To calculate the percentage of scale inhibition [R], the formula that follows was used:

1 $$\% {\text{Scale inhibition }}={\text{ }}({{\text{S}}_{{\text{in}}}} - {{\text{S}}_{\text{o}}})/{{\text{S}}_{{\text{in}}}} \times 100$$.

Where S_o_ and S_in_ represent the super saturation points without and with an inhibitor of the scale, respectively.

**Fig. 7:**
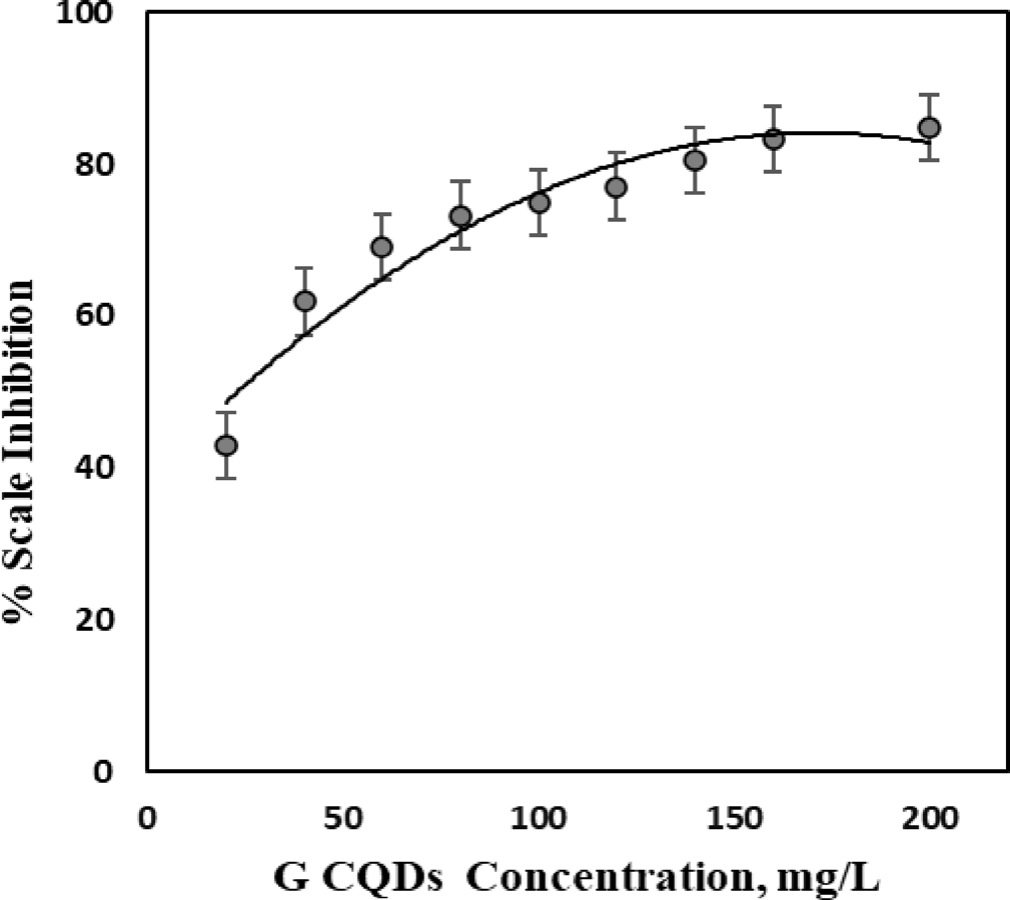
Percentage inhibition of BaSO_4_ scale formation at different G-CD concentrations.

As demonstrated in Fig. 7, the percentage of mitigation of BaSO_4_ scales rises exponentially with G-CDs, and the optimum dose was 160 mg/L, corresponding to 83.3% inhibition. This behavior may be examined based on the multiple coordination modes of G-CDs with Ba^2+^ ions because of the existences of various functional groups that are negatively charged and their adsorption at the surface of BaSO_4_ particles.

#### Adsorption behavior of G-CDs in BaSO_4_ precipitation process

**Fig. 8:**
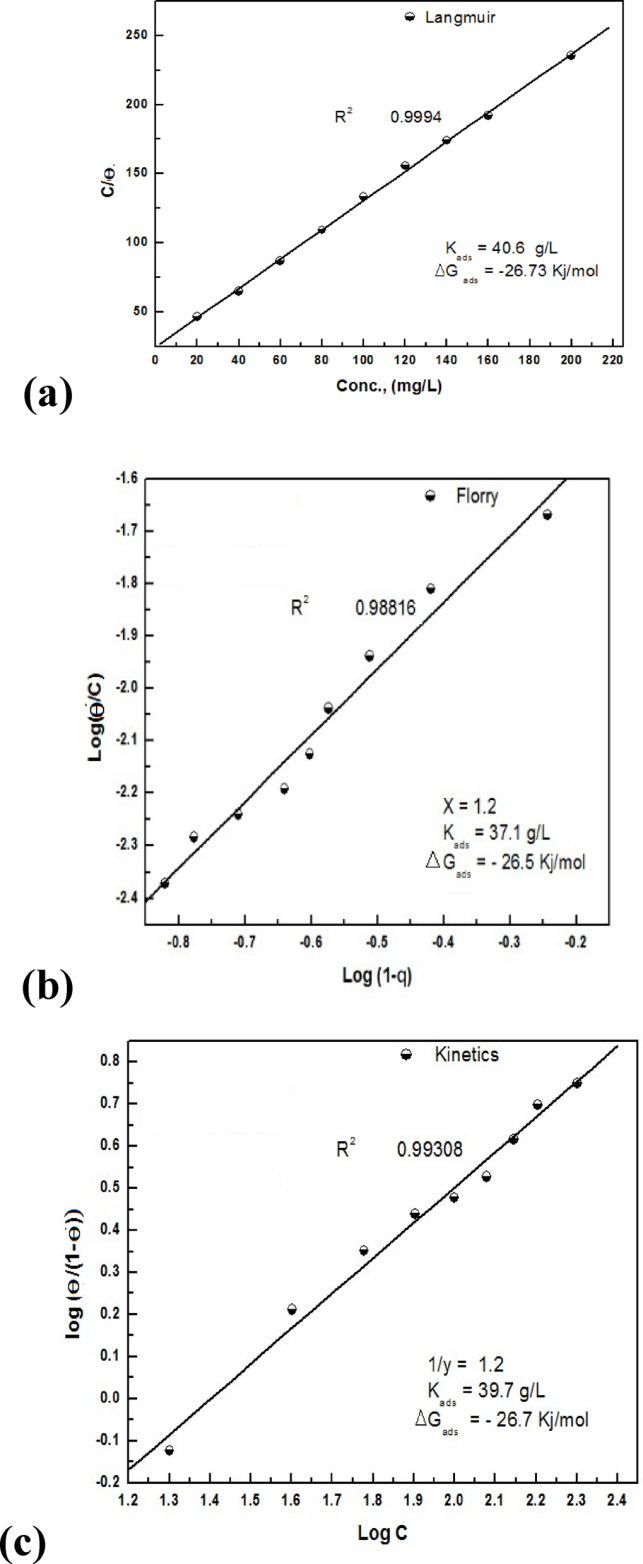
Adsorption modeling of G-CDs on BaSO_4_ crystals using: (**a**) Langmuir isotherm; (**b**) Flory–Huggins model; (**c**) Kinetic-thermodynamic model.

The assessment of G-CDs’ adsorption capacity and a clearer understanding of how adsorption behavior affects scaling inhibition efficacy are made possible by adsorption isotherms, which give crucial insights into the adsorption process. The experimental data was fitted to several adsorption isotherm models, like the kinetic-Thermodynamic model, to assess their performance. Furthermore, Flory-Huggins and Langmuir isotherms were also applied in this analysis. Langmuir adsorption isotherm Fig. 8a is assumed from Eq. (2).

2 $$\:\frac{{\text{C}}_{\text{i}\text{n}\text{h}}}{\theta}\:=\:\:\frac{1}{{\text{K}}_{\text{a}\text{d}\text{s}}}\:+\:\text{C}\text{i}\text{n}\text{h}.$$

In this context, C_inh_ ​ refers to the concentration of G-CDs in mg/L, K signifies the equilibrium adsorption constant, and θ reflects the degree of surface coverage^56^.

Figure 8b represents the Flory-Huggins isotherm using Eq. (3).

3 $$\frac{\theta }{{\left[ {x\left( {1 - \theta } \right)} \right]}}={\text{K}}\left[ {\text{C}} \right].$$

Here, x is the size factor, which indicates the number of adsorbed water molecules that a G-CDs molecule replaces^57^.

Furthermore, Fig. 8c shows the Kinetic-thermodynamic model using the formula of Eq. (4).

4 $$\:\text{L}\text{o}\text{g}\:\left[\frac{{\uptheta\:}}{(1\:-\:{\uptheta\:})}\right]=\hspace{0.17em}\text{l}\text{o}\text{g}\:{\text{K}}^{{\prime\:}}+y\:\text{log}\text{C}.$$

Whereas y represents the number of molecules from G-CDs covering a single active site, 1/y shows the number of active centers that a single G-CDs molecule occupies, and Eq. (5) gives K (binding constant).

5 $${\text{K}}\,=\,{{\text{K}}^{\prime ({\text{1 }}/{\text{ y}})}}.$$

The models demonstrated a high degree of linearity, showing the effective adhering of G-CDs on the BaSO_4_ microcrystal’s surface (Fig. 8a, b, c). The fitting degree (R²) of the models was approximately 0.999, revealing an excellent correlation. The Flory-Huggins model provided a size factor (x = 1.2), signifying that one molecule of G-CDs replaced a single water molecule on the BaSO_4_ microcrystal’s surface. In line with the kinetic-thermodynamic model, the parameter (1/y = 1.2) further confirms that each active site was occupied by one G-CD molecule. Using Eq. (6)^58^, the mentioned models produced values for ∆G°ads that were almost equal, averaging − 26.50 kJ/mol, indicating a spontaneous adsorption process.

6 $$\Delta {\text{G}}{^\circ _{{\text{ads}}}}\,=\, - \,{\text{RTln1}}000{{\text{K}}_{{\text{ads}}}}.$$

Here, (1000) indicates the water content in the solution (g/L), (R) denotes the ideal gas constant and (T) is the absolute temperature. The resulting ∆G°ads, approximately − 26.7 kJ/mol, suggests a predominantly physical cooperative adsorption process. This adsorptive behaviour of G-CDs is linked to the blockage of the active crystal growth sites of barium sulfate, and consequently, the size of crystals decreased.

#### Morphological examination of precipitated BaSO_4_ crystals

**Fig. 9:**
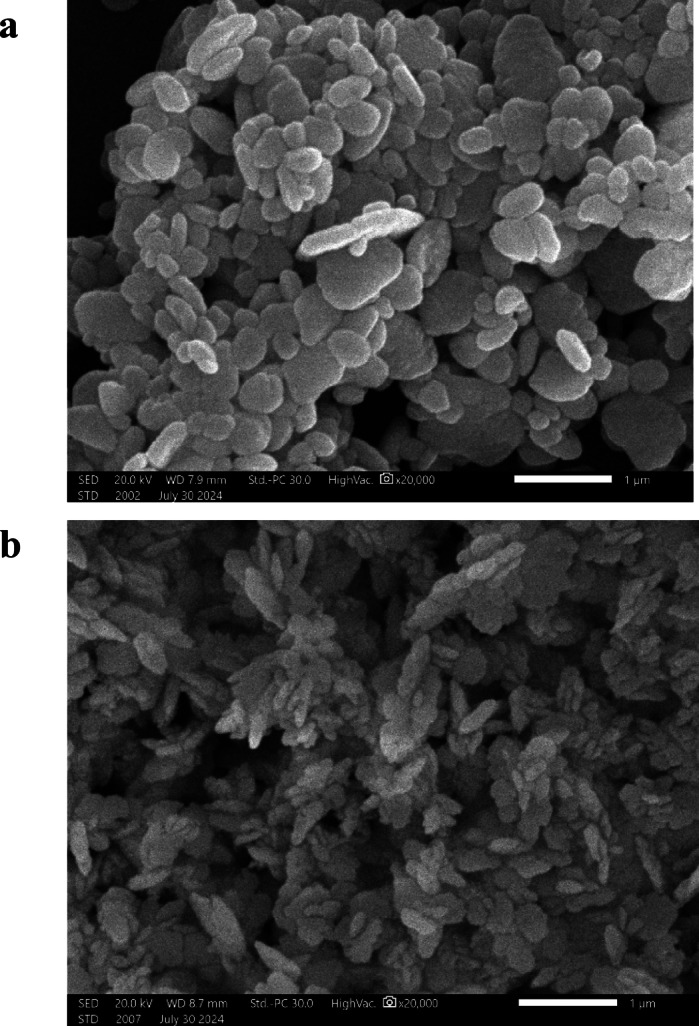
Scanning electron micrographs of BaSO_4_ crystals formed: (**a**) without G-CDs; (**b**) with G-CDs.

Figure 9 displays BaSO_4_ crystal’s SEM images after precipitation, both in the existence and absence of G-CDs. When G-CDs are absent, the crystals show irregularly shaped particles that have been termed platelet-like crystals. On the other hand, aggregates of small elongated crystals have been detected in the existence of G-CDs. Stated differently, they not only cause a decrease in crystal size but also cause morphological changes in barite crystals.

**Fig. 10:**
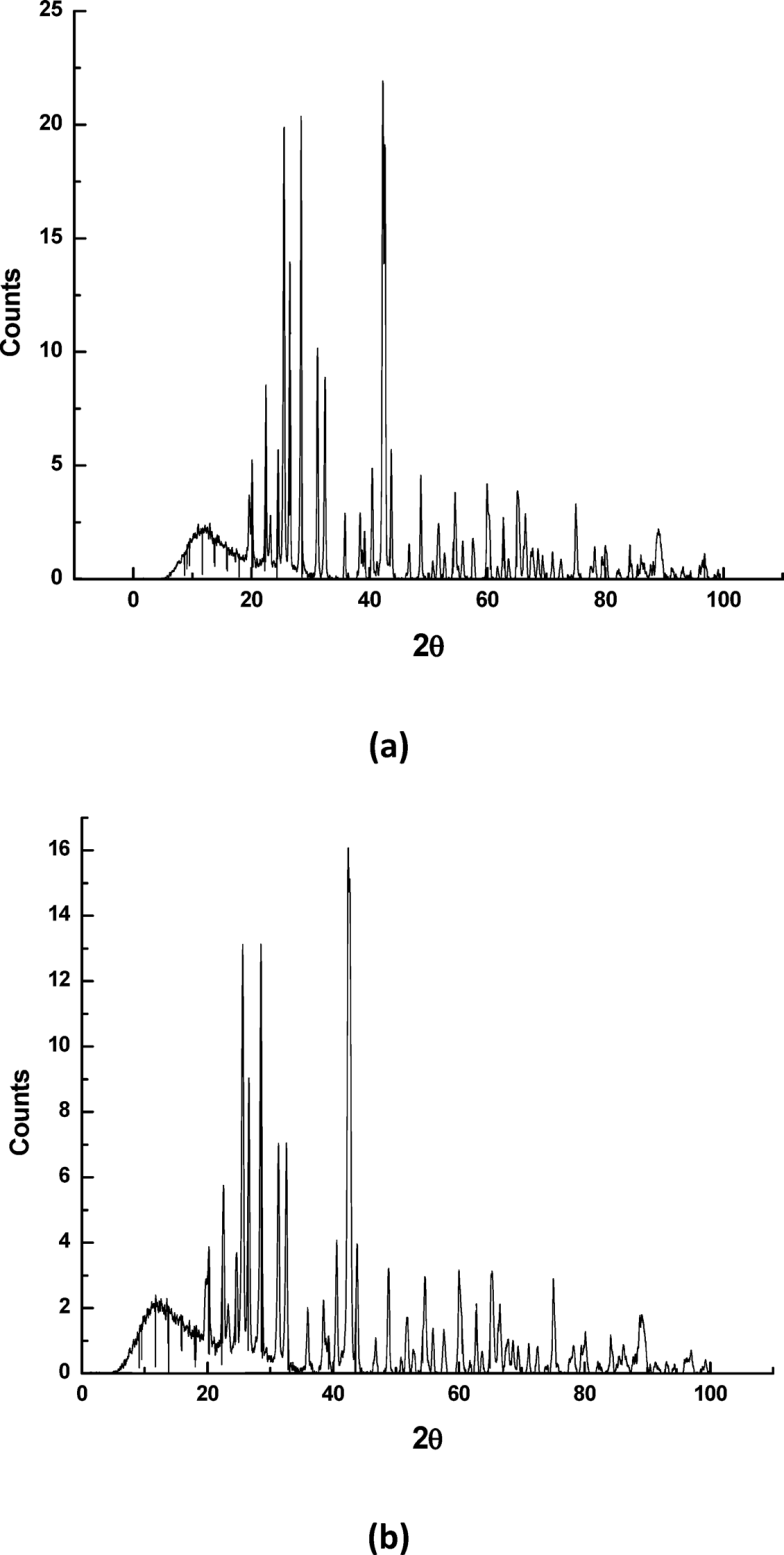
XRD patterns of BaSO_4_ precipitates: (**a**) without G-CDs; (**b**) with G-CDs.

Furthermore, XRD was employed to investigate how G-CDs affect the crystallinity of BaSO4 particles. As seen in Fig. 10(a), all diffracted lines could be indexed to BaSO_4_ structure and agree well with that reported in the literature^59^. Figure 10(b) shows that when the intensity of the various crystallographic peaks is contrasted with those when G-CDs are present, the crystallinity of the precipitated material has decreased. This behavior is explained by the G-CD molecules adhering to the surface of the BaSO4 crystals, changing their form and reducing their crystallinity.

#### Mechanism of BaSO_4_ scale Inhibition with G-CDs

Conductivity results demonstrate that G-CDs are effective, eco-friendly inhibitors of BaSO_4_ precipitation. The application of various adsorption isotherms reveals that G-CDs particles are adsorbed onto the surface of BaSO_4_ particles via a cooperative mechanism, with ∆G°ads ≃ -26.0 kJ/mol, suggesting that the adhering is predominantly physical in nature. SEM and XRD analysis of the BaSO_4_ crystals precipitated in the existence of G-CDs shows deformation of the crystal structure.

As demonstrated by FTIR spectra, G-CDs have a significant abundance of oxygen-containing groups, including carboxyl, hydroxyl, and other functional groups, distributed across their surfaces. The presence of these groups can explain the higher efficiency of G-CDs for Barium sulfate inhibition in the following ways:

Firstly, electrostatic attraction between these negatively charged groups in G-CDs to Ba^2+^ causes dynamic capturing and freeing of cations and enhances the solubility of Ba^2+^^60^. The second way is growth inhibition, which is primarily attributed to G-CDs’ interaction with barite crystal surfaces. This interaction takes place via the adsorption of charged functional groups and lone pairs in inhibitor molecules on the precipitating crystal surface, which modifies their structure and embeds further growth^61,62^. Furthermore, these functional groups can interact with calcium ions present in the media, preventing the Ba^2+^arrival at the surface of scale nuclei^63^.

### Features of corrosion Inhibition of G-CDs

To gain insight into the G-CDs impact as an inhibitor for steel corrosion in NaCl solution (0.5 M) solution, surface analysis, mass loss test, (EIS) and (PDP) are conducted.

#### Surface investigation

##### Outcomes of XRD study

**Fig. 11:**
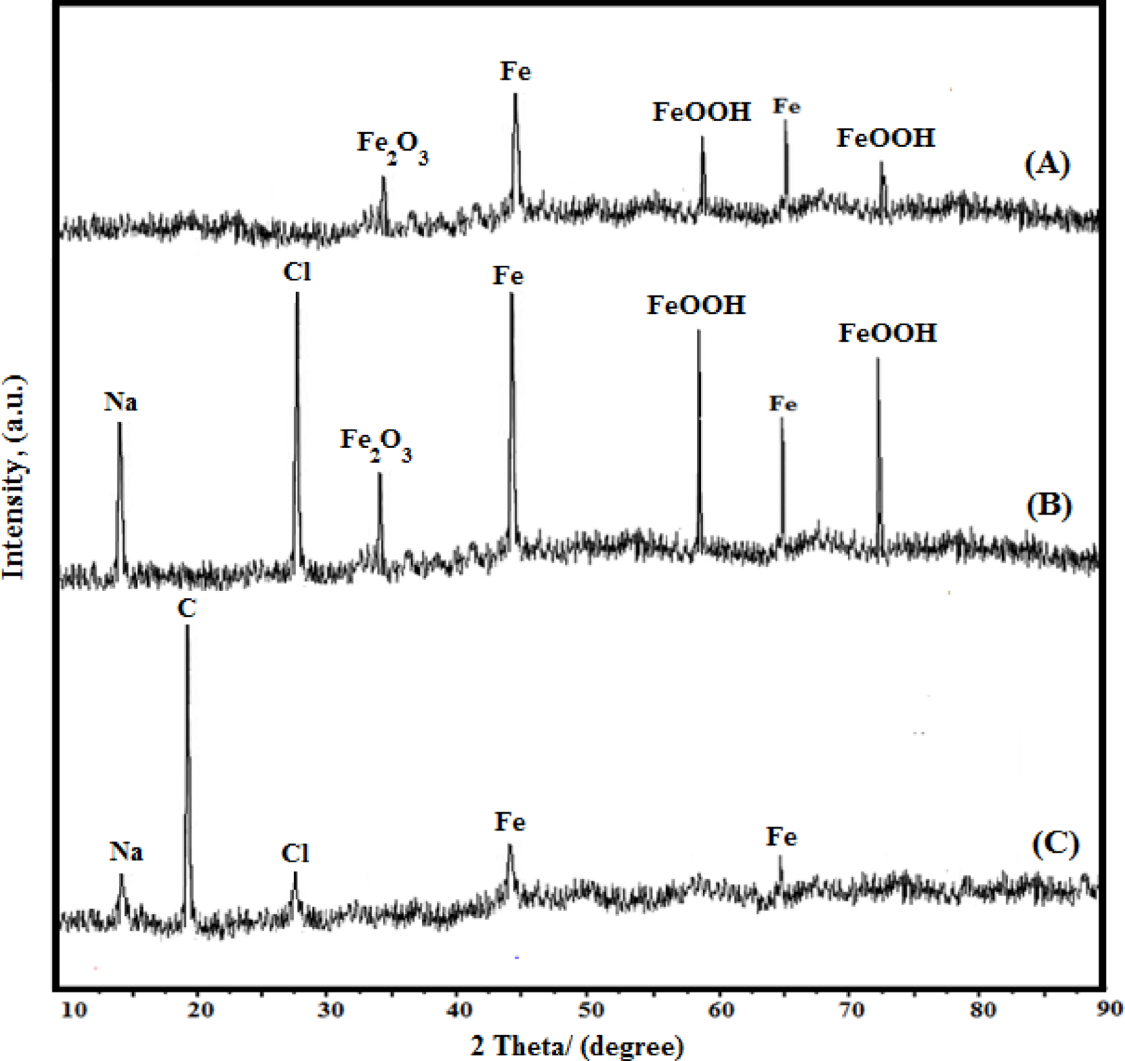
XRD patterns of corrosion products on steel after 1 h immersion: (**A**) bare steel; (**B**) steel in 0.5 M NaCl; (**C**) steel in 0.5 M NaCl with 400 mg/L G-CDs.

Figure 11 presents the XRD results for three different treatments. The carbon steel coupon’s surface that has been polished but not exposed to any treatment is shown in Fig. 11(A). Peaks of the most likely forms of oxyhydroxides (Fe_2_O_3_ and FeOOH) were found beside the peak of iron, this aligns with the literature^64^. Figure 11(B & C) illustrates the surfaces of exposed coupons immersed for 1 h in a solution of NaCl (0.5 M), the first without and the second with 400 mg/L G-CDs.

When G-CDs are present, the surface of carbon steel exhibits the peaks of iron only, while the oxides’ peaks are no longer observable. This indicates that the G-CDs completely prevented the corrosion process once they were present in the corrosion medium. Also, a carbon peak was noted at 2Ɵ° verifying the G-CDs presence on the steel’s surface. Furthermore, it is discovered that the chloride peak’s strength greatly diminishes as G-CDs was present in the corrosion medium, which can be discussed based on the displacement of specifically aggressive chloride ions with strong, surface-active and absorbable G-CDs molecules which retard the corrosion process.

##### TEM study and EDX analysis

**Fig. 12:**
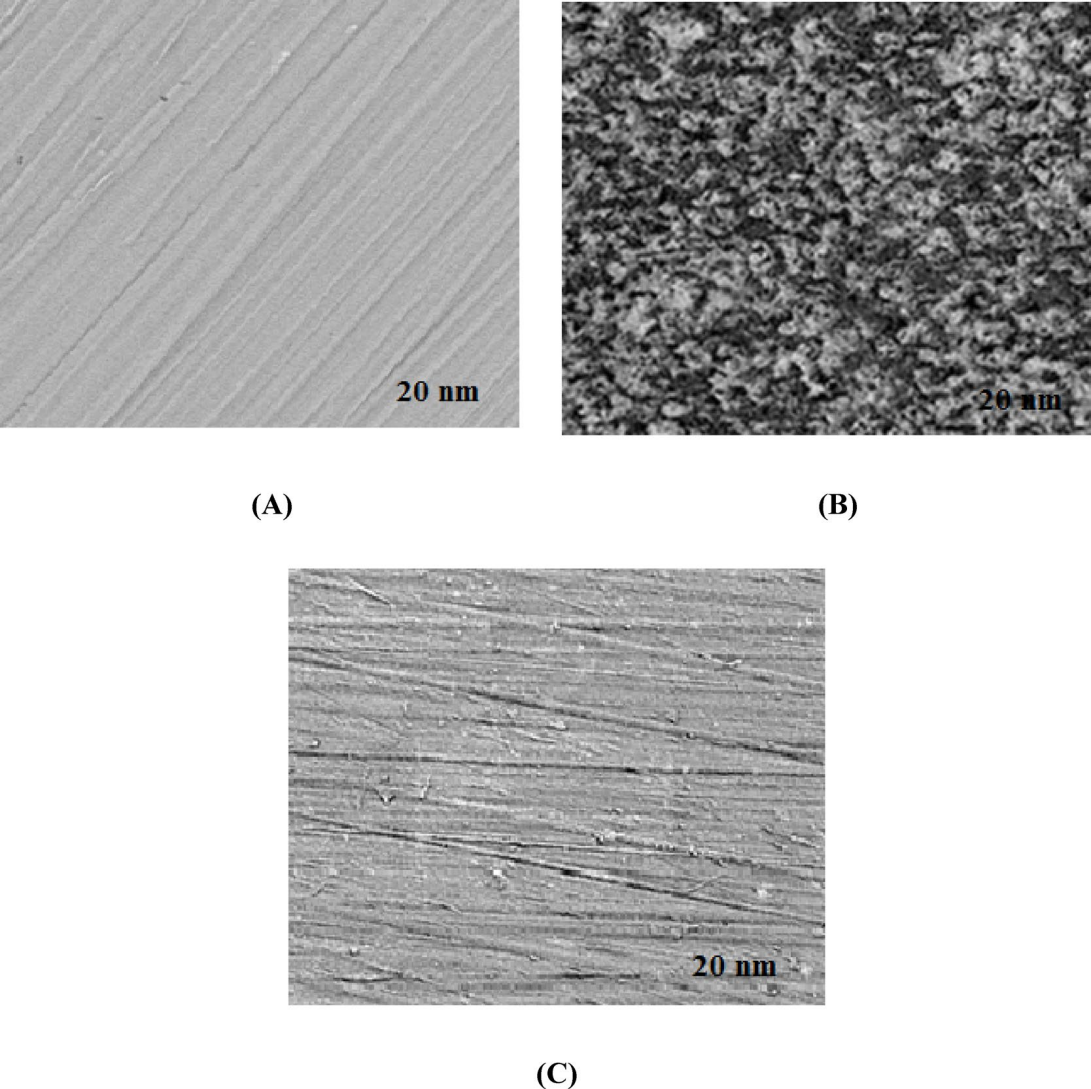
TEM images of steel surfaces: (**A**) polished steel; (**B**) steel immersed in 0.5 M NaCl for 1 h; (**C**) steel immersed in 0.5 M NaCl with 400 mg/L G-CDs.

To examine the morphology of steel’s surface subjected to three different treatments at 30 °C, TEM images were employed (Fig. 12). The TEM image labelled “A” reveals obvious polished lines on the surface of steel before immersion, demonstrating that the entire surface is uniformly smooth. Image “B” displays the steel’s surface after 1 h of submersion in a NaCl solution (0.5 M), revealing a rough and damaged texture, which indicates a strongly corrosive attack on the surface. However, after being submerged in NaCl solution (0.5 M) including 400 mg/L G-CDs, the carbon steel surface, as shown in image “C”, still displays polished markings, which indicates that G-CDs excellently mitigate the corrosion.

**Fig. 13:**
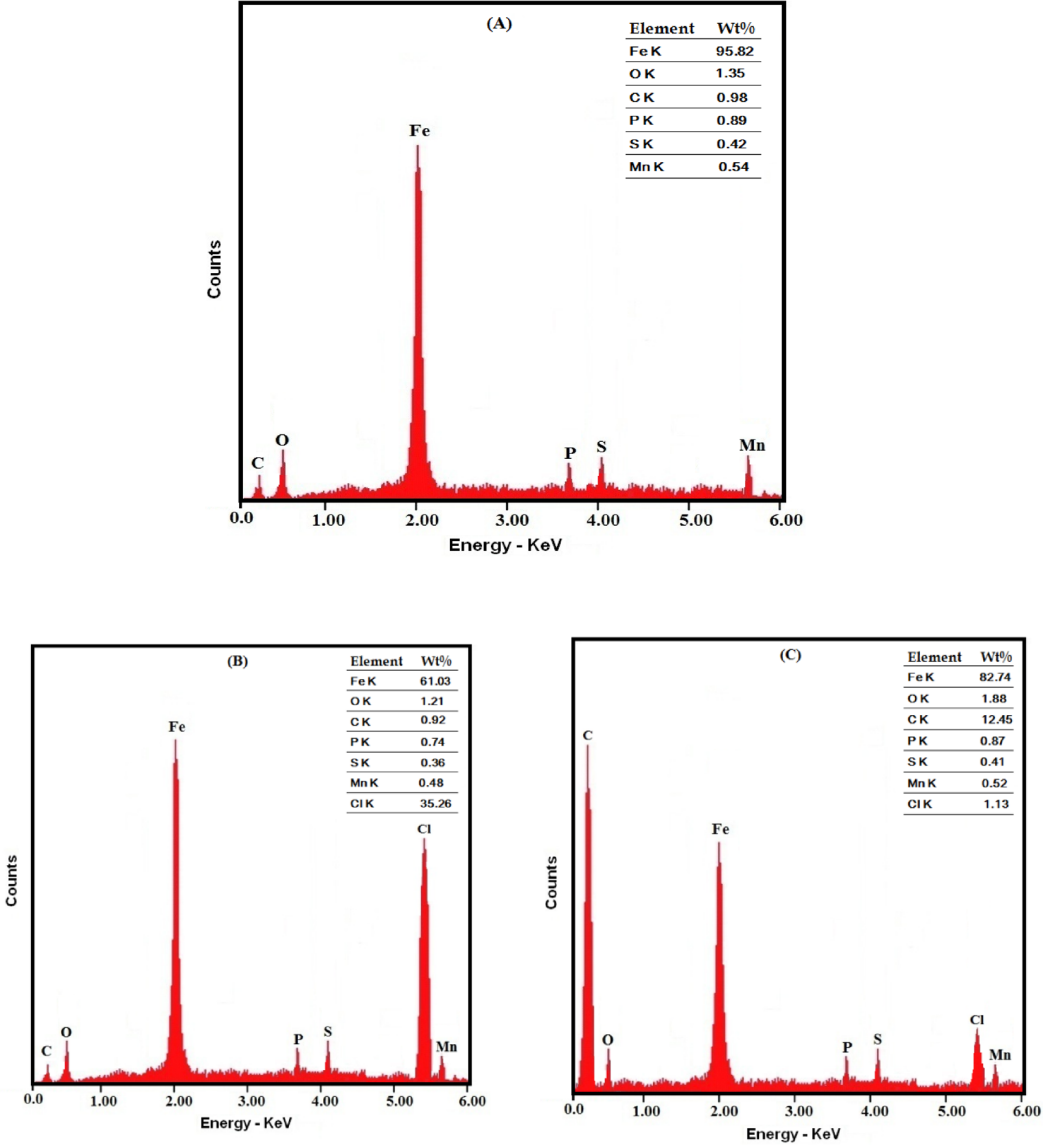
EDX micrographs of steel surfaces: (**A**) polished steel; (**B**) steel in NaCl (0.5 M); (**C**) steel in NaCl (0.5 M) + 400 mg/L G-CDs.

Additionally, the layer covering the steel surface’s chemical composition was examined employing EDX (Fig. 13). Findings distinctly revealed variations in the elemental composition across the three different treatments. The existence of the G-CDs in the corrosion medium results in: (1) Wt% of carbon was found to rise from 0.98 to 12.45, indicating that the G-CDs predominantly exist on the surface, and (2) the decrease of Wt% of chlorine from 35.25 to 1.13 indicates that the chloride ion has been displaced from the steel’s surface because of the adsorption of G-CDs. These outcomes are in line with and corroborate the XRD results.

##### XPS results

**Fig. 14:**
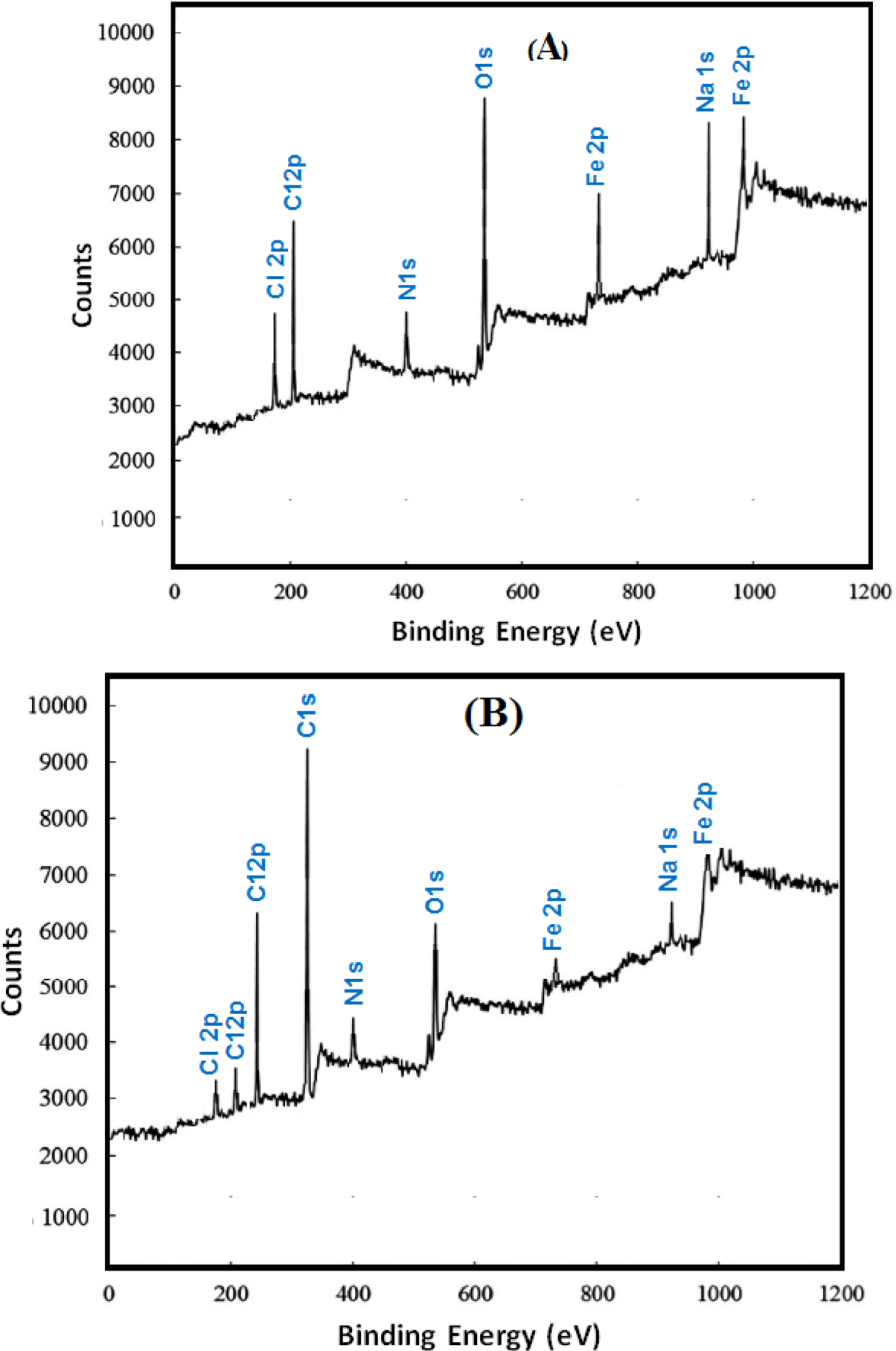
XPS spectra of steel samples after 1 h immersion: (**A**) in 0.5 M NaCl; (**B**) in 0.5 M NaCl with 400 mg/L G-CDs.

To ascertain the steel surface covering layer’s chemical composition, XPS examination was performed on two samples: one immersed in a NaCl solution (0.5 M) and the other in NaCl solution (0.5 M) with 400 mg/L of G-CDs solution (1 h at 30°C). The full survey spectra (Figs. 14A, B) provide definite proof of the adhesion of G-CDs molecules on the steel’s surface. This is verified by two key observations: 1) the prominent presence of a high-intensity C1s peak in a solution ‘B,’ which contains 400 mg/L of G-CDs, and 2) the reduction in the Cl2p peak intensity in a solution ‘B’ compared to its level in a solution of NaCl (0.5 M) alone. These outcomes suggest that steel is protected from corrosion in a 0.5 M NaCl solution because of the effective adhesion of G-CDs on the steel surface, along with the desorption of chloride ions. This indicates that the highly aggressive chloride ions are being replaced by strongly surface-active, absorbable G-CD molecules.

Additionally, the diminished peak intensities of Fe and O on the steel surface following immersion in solution ‘B’ further confirm that G-CDs reduce the formation of corrosion products, effectively hindering the corrosion process.

##### Measurements of contact angles

**Fig. 15:**
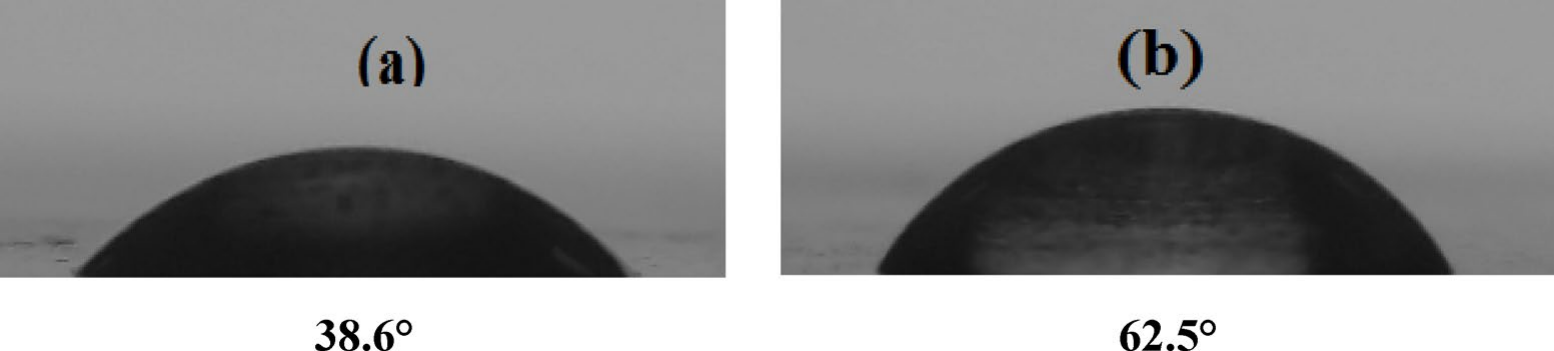
Contact angle measurements on steel: (**a**) after immersion in 0.5 M NaCl; (**b**) with 400 mg/L G-CDs.

The protective layer formation on carbon steel surfaces was confirmed by contact angle measurements. Figure 15 shows contact angle measurement images of two samples: one that was submerged in a NaCl solution (0.5 M) (Fig. 15a) and the other that was immersed in a NaCl solution (0.5 M) including 400 mg/L of G-CDs (Fig. 15b). The first sample’s contact angle (Fig. 15a) was found to be 38.6°. Conversely, the second one (Fig. 15b) saw a rise in contact angle to 62.5°. This change indicates that the interfacial hydrophobicity was greatly increased and the wettability was decreased upon G-CDs adhering to the steel surface. The finding hydrophobic covering layer successfully prevented Cl ions from permeating the metal substrate and preventing corrosion.

#### Weight loss results

**Fig. 16:**
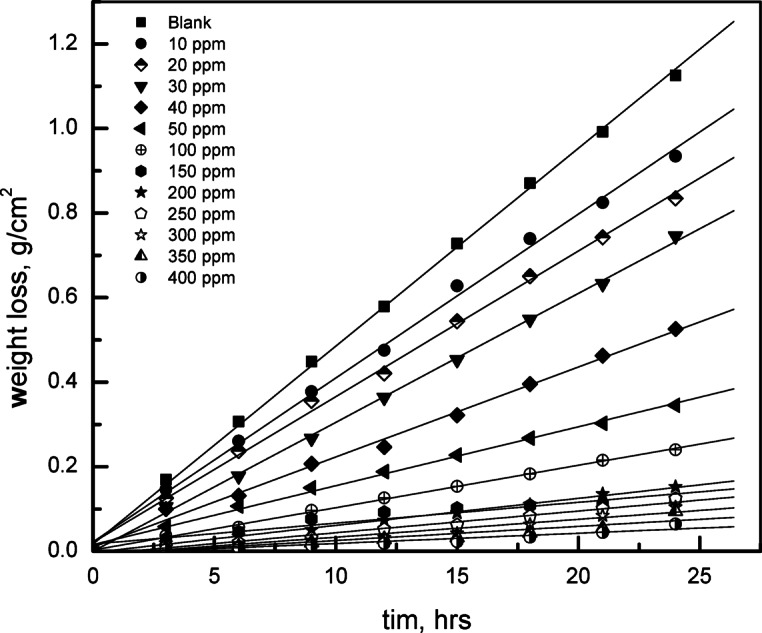
Time-dependent weight loss of carbon steel at 30 °C in 0.5 M NaCl with varying G-CD concentrations.

Figure 16 displays the time-dependent weight loss data at 30 °C for carbon steel in a NaCl solution (0.5 M). As the concentration of G-CDs increased, the rate of corrosion considerably dropped in comparison to the blank solution and finally stabilized at its lowest value at 400 mg/L. This demonstrates the effective protective impact of G-CDs. The (% p) inhibitory effectiveness of G-CDs was determined using Eq. (7) and is shown in Table 1^65^.

7 $$\% {\text{P}}={\text{ }}[({{\text{R}}_{\text{o}}}--{\text{R}})/{{\text{R}}_{\text{o}}}]{\text{ x1}}00$$.

Where R and R_o_ reflect the corrosion rates with and without the presence of G-CDs, respectively, which are reported in g/cm^2^.hr-^1^.

**Table 1:** Data from weight loss of carbon steel coupons at 30 °C in a NaCl solution (0.5 M) with various G-CD concentrations at 24 h.

Conc.(mg/L)	Rate ( g.cm^− 2^.hr^− 1^)	% *P*
0	0.04682 ± 0.19	--
10	0.03779 ± 0.17	19.3 ± 0.21
20	0.03447 ± 0.16	26.4 ± 0.23
30	0.03052 ± 0.15	34.8 ± 0.26
40	0.02430 ± 0.13	48.1 ± 0.28
50	0.01896 ± 0.11	59.5 ± 0.29
100	0.01302 ± 0.09	72.2 ± 0.24
150	0.00894 ± 0.08	80.9 ± 0.31
200	0.00639 ± 0.06	86.4 ± 0.34
250	0.00514 ± 0.05	89.0 ± 0.27
300	0.00422 ± 0.04	90.9 ± 0.35
350	0.00329 ± 0.03	92.9 ± 0.37
400	0.00273 ± 0.02	94.2 ± 0.39

#### Electrochemical assessment

##### EIS evaluation

**Fig. 17:**
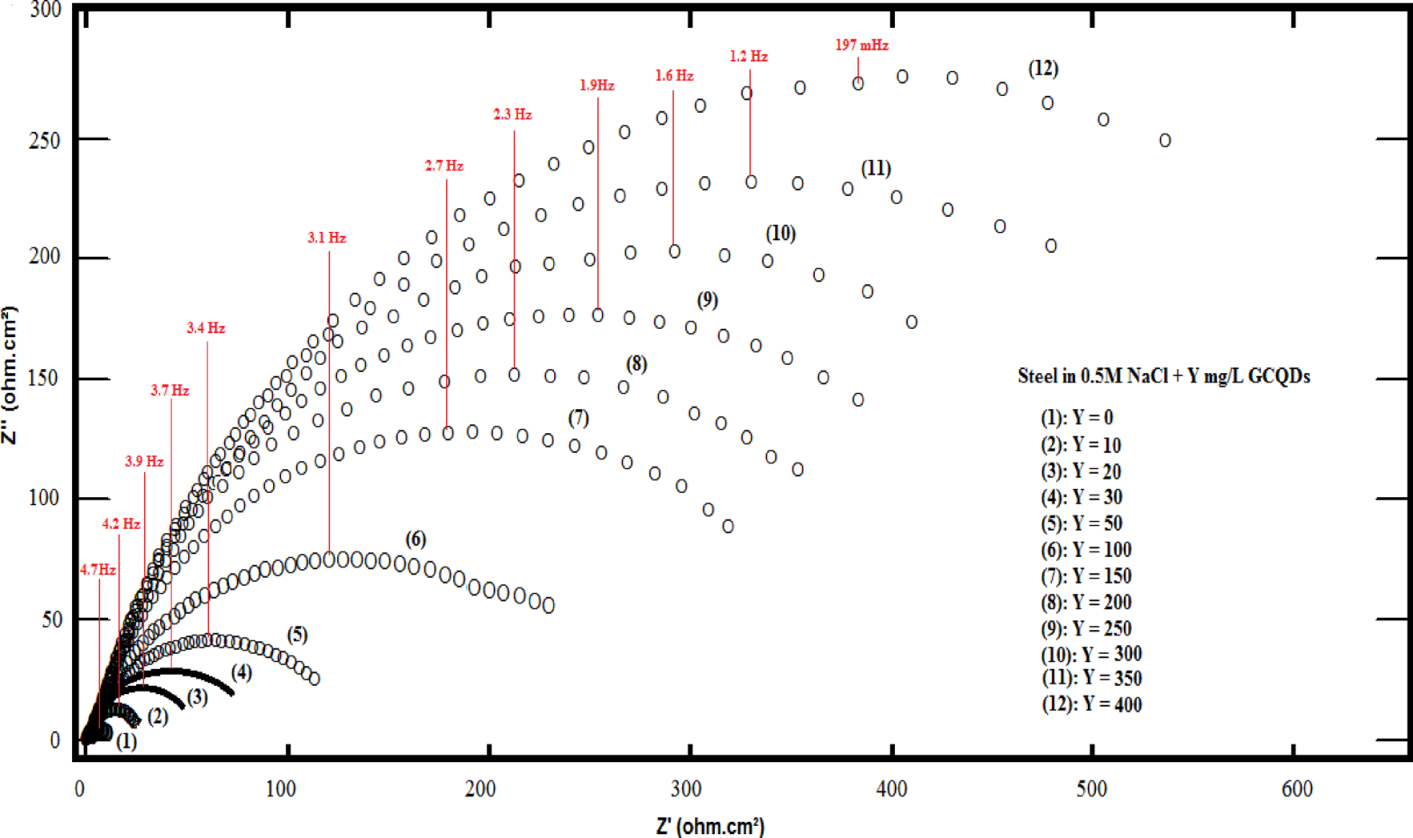
Nyquist plots of carbon steel in 0.5 M NaCl with and without varying doses of G-CDs.

Figure 17 offers the Nyquist plots for carbon steel in a NaCl solution (0.5 M), both in the absence and presence of varying concentrations of G-CDs. The findings reveal only a single depressed capacitive semicircle. The deviation from perfect semicircles in the Nyquist plots can be attributed to factors such as surface roughness, the presence of impurities, electrode heterogeneity, and the adsorption of the inhibitor, which leads to the formation of a porous layer that progressively renders the electrode surface more uniform^66^. Semicircles get larger in diameter when the concentration of G-CDs increases, suggesting that higher concentrations of the G-CDs can substantially mitigate the corrosion induced by the aggressive solution. This can be clarified through the ability to effectively cover a larger corrosion-affected area with higher concentrations of the inhibitor, improving the protection against corrosion and producing an adhering film on the surface of steel. Furthermore, the consistent shape of all Nyquist curves suggests that the inhibitor’s presence raised the corrosion system’s impedance value without changing other electrochemical characteristics^35^.

**Fig. 18:**
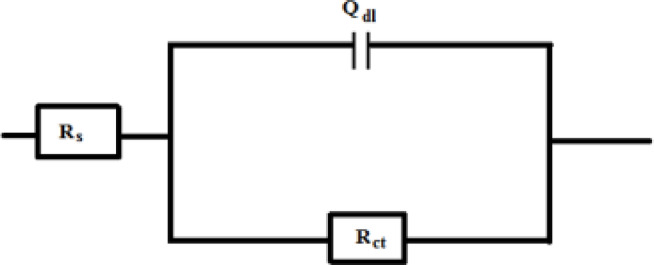
Equivalent circuit model used for fitting (EIS) data.

The corresponding circuit is shown in Fig. 18, and Zsimpwin was used to analyze all EIS data. This circuit comprises the charge transfer resistance (R_ct_), the solution resistance (Rs) and a constant phase element (Q). A strong correlation was seen between the fitted and measured spectra by using the equivalent circuit model. The measurements and parameters had a high degree of precision, with an inaccuracy of less than 3%. Using Eq. (8), the inhibition efficiency was calculated^67^.

8 $$\% {\text{P }}={\text{ }}[({{\text{R}}_{{\text{ct}}}}--{{\text{R}}^{\text{o}}}_{{{\text{ct}}}})/{{\text{R}}_{{\text{ct}}}}]{\text{ x1}}00$$.

Given that, respectively, R_ct_ and R^o^_ct_ are the resistance of charge transfer with and without G-CDs.

The findings for EIS parameters are manifested in Table 2. The Rs values showed a gradual increase due to the partial blocking of active corrosion sites and reduced electrolyte access to the metal surface. However, the addition of the G-CDs led to an increase in the R_ct_ value, with this impact becoming more pronounced at higher concentrations. This suggests that the G-CDs effectively slow down the charge transfer process during corrosion. The Q_dl_ is used in the equivalent circuit to account for the non-ideal capacitive behavior of the electrochemical double layer. The non-ideal double-layer capacitance Q_dl_ is numerically equal to the admittance (Y_0_). The Y_0_ component of the constant phase element (CPE) reflects various physical processes such as the formation of porous layers, inhibitor adsorption, and surface inhomogeneities caused by surface roughness^68^. The values of Q_dl_ drop with increasing G-CDs dose. This is mostly caused by an increase in the thickness of the electric double layer and a decrease in the dielectric constant, and this confirms the effective adsorption of G-CDs on the steel surface^69^. The interfacial ideal double-layer capacitance (C_dl_) can be calculated from the constant phase element (CPE) parameters (Y_0_and n) using Eq. (9)^70^.

9 $${{\text{C}}_{{\text{dl}}}}={({{\text{Y}}_0} \times {{\text{R}}_{{\text{ct}}}})^{{\text{1}}/{\text{n}}}}/{{\text{R}}_{{\text{ct}}}}$$.

The n value in the CPE (where − 1 ≤ *n* ≤ 1) reflects the degree of deviation from ideal capacitive behavior:

- *n* = 1 represents an ideal capacitor, indicating a perfectly homogeneous surface.
- *n* = -1 represents an ideal inductor,
- *n* = 0.5 represents Warburg diffusion behavior, typically associated with diffusion-controlled processes.
- 0 < *n* < 1 suggests a non-ideal capacitive behavior, often attributed to surface heterogeneity, roughness, or a distribution of relaxation times at the electrode/electrolyte interface.

In the present study, n remains constant at 0.9 across all concentrations of G-CDs, including the blank sample, indicating that the surface maintains a relatively homogeneous and smooth capacitive character even after G-CD adsorption. This high and constant n value (close to 1) suggests:

1. **Minimal Surface Roughness Effects**: Even though surface modification is occurring due to G-CD deposition, the surface remains electrochemically uniform, with negligible additional roughness or porosity.
2. **Efficient Film Formation**: The constancy of n supports the idea that G-CDs form a compact and homogenous inhibitive layer on the steel surface, enhancing corrosion resistance without introducing significant irregularities.

The chi-squared (χ²) values were used to assess the accuracy of the fitting results. The low χ² values obtained (Table 2) indicate an excellent agreement between the experimental data and the fitted curves^71,72^. As noted in the literature, the inhibitor molecules interact with the steel surface, displacing H_2_O molecules at the steel/solution interface^73^. Furthermore, when G-CDs concentration increased, the corrosion inhibition efficacy (% P) improved. As the efficiency of corrosion inhibition reached 98.3% at 400 mg/L.

**Table 2:** The electrochemical parameters discovered from Nyquist curves.

Conc., mg/L	*R*_s_ (Ohm.cm^2^)	Q_dl_ (µF)	*n*	C_dl_ µFcm^− 2^	*R*_ct_ (Ohm.cm^2^)	% *P*	Goodness of fit (χ^2^)
**0**	0.02± 0.001	1243	0.9 ± 0.01	3615.8	12 ± 0.05	--	9.8 × 10^− 5^
**10**	0.03 ± 0.001	1208	0.9 ± 0.01	3664.3	18 ± 0.07	33.3 ± 0.11	9.7 × 10^− 5^
**20**	0.05 ± 0.003	1168	0.9 ± 0.01	3677.0	26 ± 0.09	53.8 ± 0.16	9.6 × 10^− 5^
**30**	0.06 ± 0.002	1104	0.9 ± 0.01	3558.3	34 ± 0.11	64.7 ± 0.21	9.5 × 10^− 5^
**40**	0.07 ± 0.001	1042	0.9 ± 0.01	3541.0	58 ± 0.13	79.3 ± 0.24	9.2 × 10^− 5^
**50**	0.09 ± 0.001	987	0.9 ± 0.01	3483.1	86 ± 0.15	86.0 ± 0.27	8.7 × 10^− 5^
**100**	0.11 ± 0.003	862	0.9 ± 0.01	3228.0	168 ± 0.16	92.8 ± 0.31	7.6 × 10^− 5^
**150**	0.12 ± 0.002	748	0.9 ± 0.01	2941.8	301 ± 0.18	96.0 ± 0.35	5.6 × 10^− 5^
**200**	0.14 ± 0.002	630	0.9 ± 0.01	2502.0	390 ± 0.19	96.9 ± 0.39	4.3 × 10^− 5^
**250**	0.16 ± 0.003	552	0.9 ± 0.01	2183.8	430 ± 0.22	97.2 ± 0.43	3.8 × 10^− 5^
**300**	0.18 ± 0.001	488	0.9 ± 0.01	1927.7	480 ± 0.25	97.5 ± 0.46	3.0 × 10^− 5^
**350**	0.19 ± 0.001	337	0.9 ± 0.01	1308.5	595 ± 0.27	97.9 ± 0.48	1.4 × 10^− 5^
**400**	0.21 ± 0.001	287	0.9 ± 0.01	1112.4	688 ± 0.28	98.3 ± 0.51	1.0 × 10^− 5^

##### Potentiodynamic polarization outcomes (PDP)

**Fig. 19:**
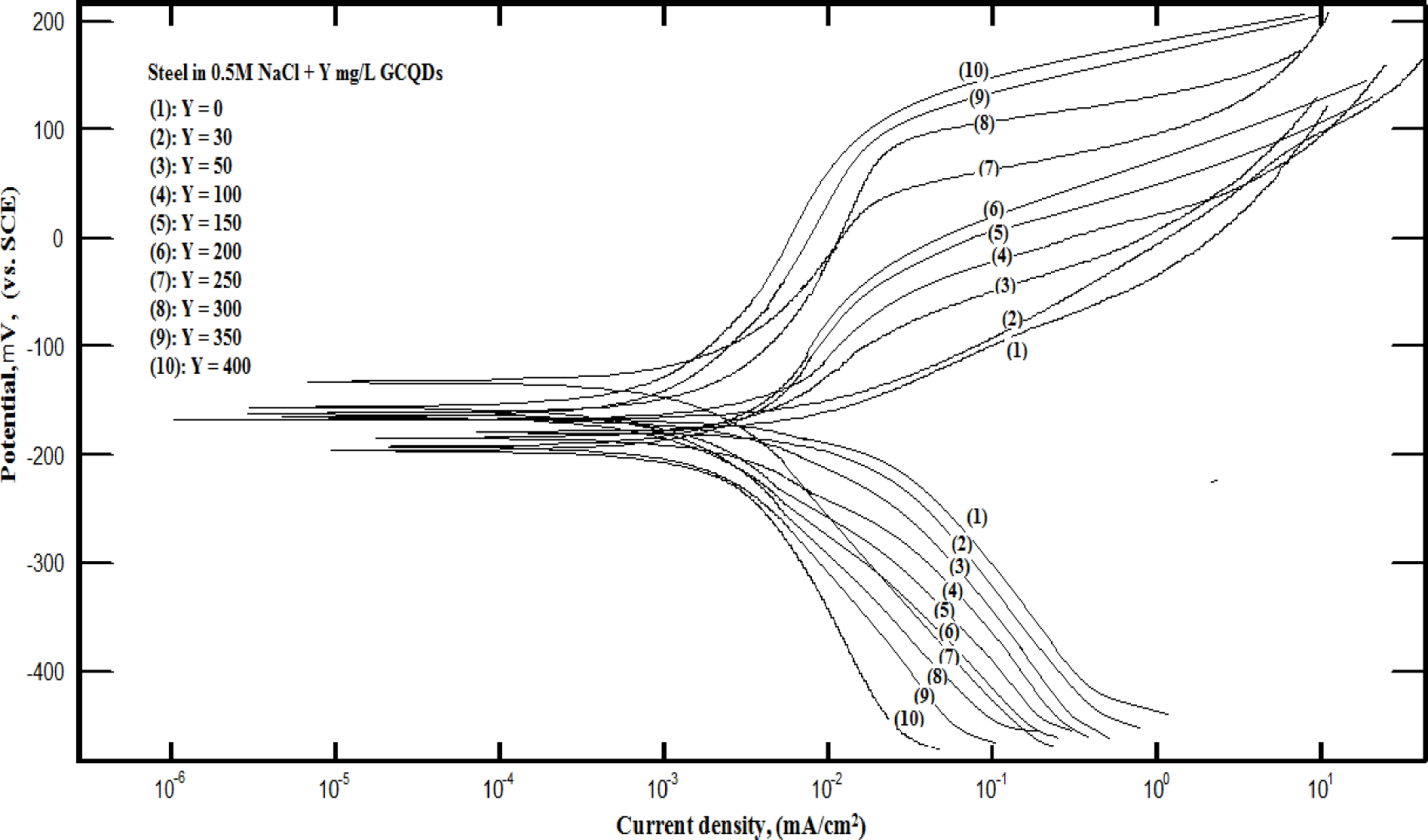
Potentiodynamic polarization (PDP) curves of carbon steel submerged in a NaCl solution (0.5 M) with and without varying doses of G-CDs.

PDP curves were employed to examine the anodic and cathodic processes and evaluate the effectiveness of G-CDs in inhibiting these processes. Figure 19 displays the PDP carbon steel curves in a NaCl solution (0.5 M) without and with different doses of G-CDs. The associated variables are detailed in Table 3, obtained by classical extrapolation of the Tafel regions of the PDP curves. Among these, *icorr*,* Ecorr*, *βc*, *β*a, and P stand for corrosion current density, corrosion potential, cathodic Tafel slope, anodic Tafel slope, and inhibition efficiency, respectively. These graphs demonstrate how G-CDs operate as a mixed inhibitor. The data indicates that a rise in the concentration of G-CDs causes a change in both the anodic and cathodic curves towards lower *i*_corr_.

The formula yields the P value:

9 $$\% {\text{P }}={\text{ }}\left[ {\left( {{i_{{\text{o corr}}}}} \right){\text{ }} - {\text{ }}\left( {{i_{{\text{corr}}}}} \right){\text{ }}/{\text{ }}\left( {{i_{{\text{o corr}}}}} \right)} \right]{\text{ x 1}}00$$.

Where *i*_o corr_ and *i*_corr_ stood for the inhibited and uninhibited steel’s respective current densities^65^.

**Fig. 20:**
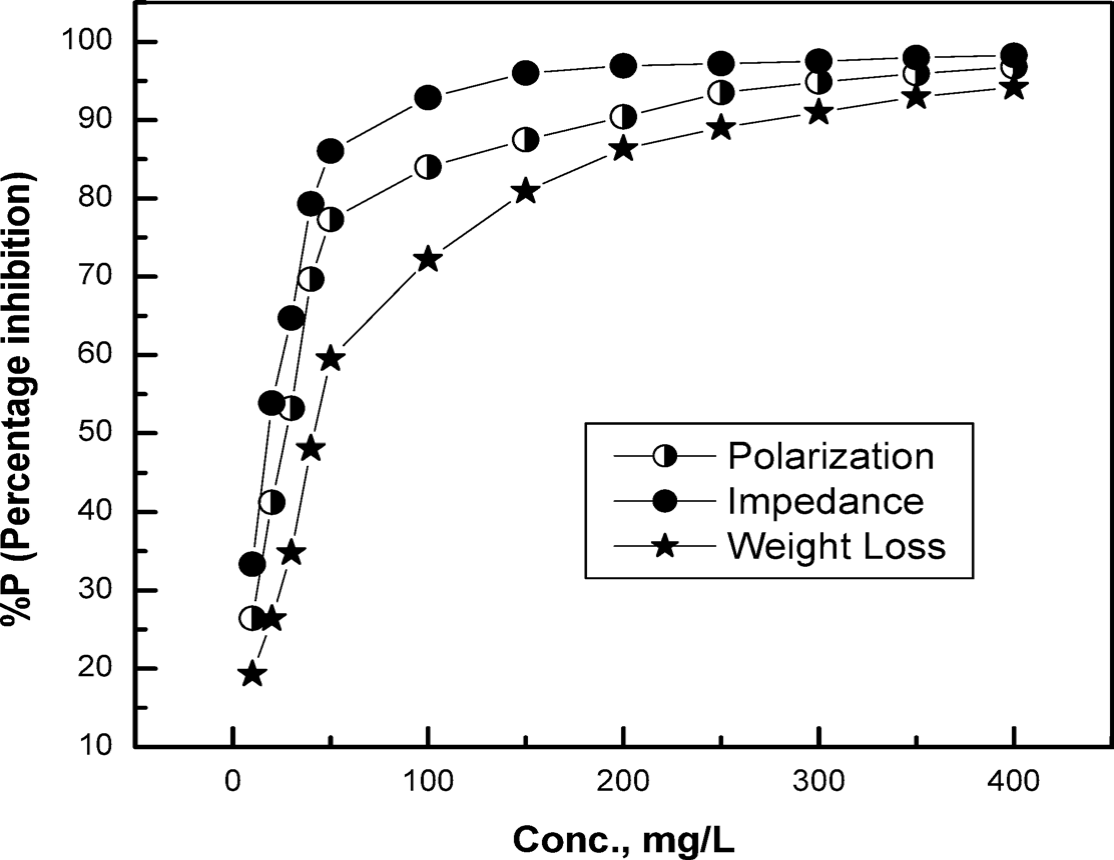
Variation in corrosion inhibition efficiency (%P) of G-CDs based on PDP, EIS, and weight loss results at different concentrations.

Table 3 illustrates that the addition of G-CDs findings in a decline in *βc* and a rise *in βa* values, as well as a decrease in *i*_*corr*_ relative to the blank in NaCl solution (0.5 M). This shows that G-CDs function as an inhibitor of mixed types, significantly hindering anodic and cathodic processes^74^. It should be noted that when the concentrations of G-CDs rise, the *i*_*corr*_ value significantly exhibits a downward trend. In accordance with this, the % P value of G-CDs shows an increasing trend and reaches 96.8% at a maximum G-CD of 400 mg/L concentration. This effect is likely due to the effective protective layer that the G-CDs adsorption creates. These findings align well with the outcomes of the mass loss test and EIS seen in Fig. 20.

**Table 3:** Electrochemical characteristics derived from PDP curves.

Conc., mg/L	-E_corr_ (mV)	B_a_ mV/decade	- B_c_ mV/decade	i_corr_ (mA/cm^2^)	% *P*
0	205 ± 1.08	92 ± 0.51	126 ± 0.91	3316 ± 1.74	--
10	201 ± 1.11	89 ± 0.49	128 ± 0.87	2808 ± 1.38	26.4 ± 0.26
20	197 ± 1.10	86 ± 0.46	132 ± 0.85	2242 ± 1.35	41.2 ± 0.29
30	194 ± 1.18	83 ± 0.42	136 ± 0.82	1787 ± 1.29	53.2 ± 0.31
40	191 ± 1.15	76 ± 0.39	139 ± 0.79	1157 ± 1.28	69.7 ± 0.34
50	188 ± 1.21	71 ± 0.35	141 ± 0.76	866 ± 1.25	77.3 ± 0.37
100	180 ± 1.48	62 ± 0.31	145 ± 0.74	610 ± 1.33	84.0 ± 0.41
150	174 ± 1.26	58 ± 0.27	148 ± 0.71	478 ± 1.23	87.5 ± 0.57
200	137 ± 1.28	54 ± 0.25	151 ± 0.68	366 ± 1.21	90.4 ± 0.46
250	168 ± 1.25	51 ± 0.23	153 ± 0.65	248 ± 1.18	93.5 ± 0.49
300	162 ± 1.30	48 ± 0.19	157 ± 0.61	196 ± 1.16	94.9 ± 0.52
350	155 ± 1.33	45 ± 0.16	163 ± 0.57	156 ± 1.13	95.9 ± 0.55
400	151 ± 1.34	42 ± 0.11	167 ± 0.55	123 ± 1.12	96.8 ± 0.59

#### Adsorption isotherm evaluation

**Fig. 21:**
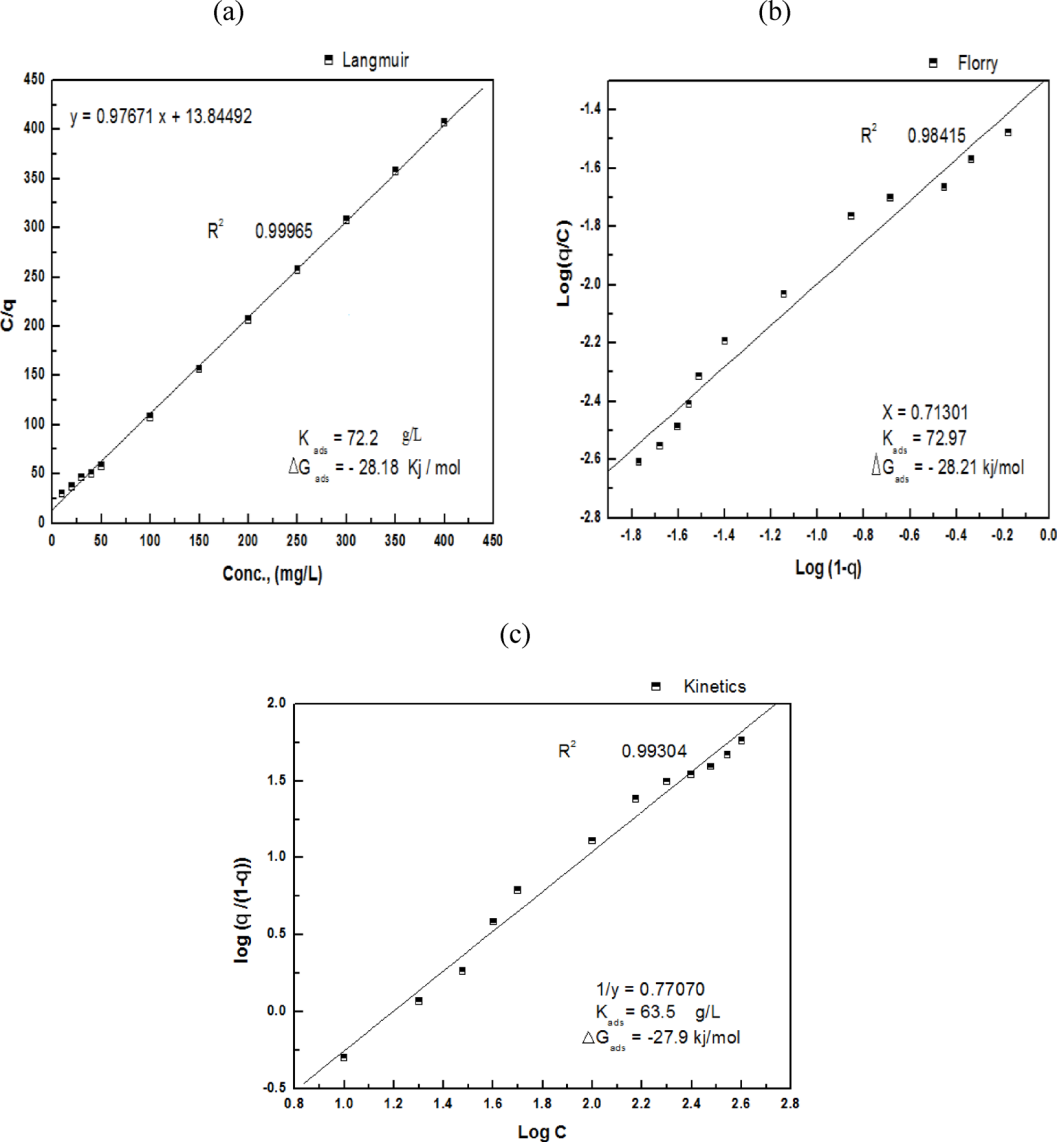
Adsorption modeling of G-CDs on steel surface using EIS data: (**a**) Langmuir isotherm; (**b**) Flory–Huggins model; (**c**) Kinetic-thermodynamic model.

To better comprehend the G-CD adsorption isotherms on the surface of the steel, numerous adsorption isotherm models, including the kinetic-thermodynamic model along with Flory-Huggins and Langmuir isotherm, were applied to analyze and fit the outcomes obtained from the EIS plots. By using the Eqs. 2,3,4,5 and 6 in Sect. 3.2.3, the results were calculated and documented in Table 4. Figure 21 demonstrates a strong linear correlation for the mentioned models, suggesting that the G-CDs adsorption on the carbon steel surface in a NaCl solution (0.5-M) is an ideal process.

The size factor (x = 0.7) represents the fact that one molecule of H_2_O from the surface was replaced by one molecule of G-CDs. As stated in the Kinetic-thermodynamic model, with 1/y = 0.7, a single G-CD molecule occupies each active site. The resultant ∆G^o^_ads_ ≃ -28.0 kJ/mole suggests a predominantly physical cooperative adsorption process. This adsorptive behavior of G-CDs is connected with the higher efficiency of blockage of the steel surface’s active sites, consequently hindering the steel corrosion in a NaCl solution (0.5 M).

**Table 4:** Linear characteristics that result from using adsorption models.

Langmuir		Flory- Huggins			Kinetic-Thermodynamic		
K	-∆G⁰_ads_ (kJ/mol)	K	-∆G⁰_ads_ (kJ/mol)	X	K	-∆G⁰_ads_ (kJ/mol)	1/y
72.2	28.18	72.97	28.21	0.7	63.5	27.9	0.7

#### Mechanism of corrosion Inhibition with G-CDs

The steel surface was studied by XRD, EDX and XPS techniques after immersion for one hour in a NaCl solution (0.5-M) both without and with 400 mg/L G-CDs. the findings Highlighted that the existence of carbon quantum dots in the corrosion chloride medium resulted in (1) strong G-CDs particles adhering to the surface of steel and (2) significant chloride ion desorption from the steel surface.

These phenomena can be discussed on the basis that:

##### The anodic corrosion reaction

Fe →Fe^2+^+2e^−^.

##### The cathodic corrosion reaction

H_2_O + 1/2O_2_ + 2e^−^→2OH^−^.

In the absence of the carbon quantum dots in the chloride medium, the Cl⁻ ions are specifically adsorbed on the steel surface and catalyse both the charge transfer cathodic and anodic reactions. However, in the presence of the G-CDs in the environment, the highly surface-active, strong adsorbable quantum dot particles displace the Cl⁻ ions from the steel surface, causing mitigation of the catalytic effect, which leads to the inhibition of the corrosion process.

## Conclusion

The investigation’s conclusions show that:

1. Using a one-step pyrolysis technique, G-CDs (Ginger Carbon Quantum Dots) were effectively created, with a 3–6 nm average particle size. XRD, EDX, FTIR, and XPS analyses revealed that G-CDs are primarily made up of carbon, oxygen, and nitrogen, with a significant amount of unsaturated double bonds.
2. The scale inhibition behavior of G-CDs was studied using the conductivity technique; the results revealed that the synthesized G-CDs have an inhibition efficiency of 84.6% at 200 mg/L.
3. XRD and SEM examinations of the BaSO_4_ crystals after precipitation in existences of 160 mg/L showed deformation of the crystals.
4. Kinetic-thermodynamic model along with Langmuir and Flory-Huggins adsorption isotherms, were utilized to fit the experimental findings. The results confirmed the applicability of both isotherms and models, yielding a ∆G° _precipitation_ ≃ 26.7 kJ/mol. This indicates that the adhering of G-CDs onto the BaSO_4_ crystals surface occurs through a cooperative mechanism (physical/chemical) primarily physical.
5. EIS and the mass loss method were employed to investigate the G-CDs corrosion mitigation properties. The outcomes revealed that G-CDs work as a superior eco-friendly anti-corrosion agent for steel, achieving 98.3% inhibition effectiveness at 400 mg/L in a NaCl solution (0.5 M). However, the PDP results revealed that the G-CDs act as mixed-type inhibitors.
6. The adsorption isotherms are applied to fit the experimental corrosion inhibition data and the three isotherms are applicable giving (∆G°_ads_) values equal to − 28.2 kJ/mol indicating that the adsorption process of the G-CDs on the steel surface is cooperative (physical/chemical).
7. The surface studies XPS, XRD, TEM, and EDX of the steel surface after submerged in a NaCl solution (0.5 M) for 1 h revealed that the G-CDs are strongly adsorbed and the aggressive Cl^−^ ions are desorbed from the steel surface causing corrosion retardation.
8. A mechanism of the inhibition of the corrosion of steel in chloride media by G-CQDs was presented and the results suggest that in absence of the carbon dots the Cl^−^ ions are specific adsorbed on the steel surface and catalyse the charge transfer cathodic and anodic corrosion reactions. However, in presence of the carbon dots in the environment the strong active absorbable carbon dots particles displace the Cl^−^ ions from the steel surface mitigating the catalyse effect of the cathodic and anodic corrosion reaction which leads to the inhibition of the corrosion process.

## Data Availability

Data used or analyzed during the current study available from the corresponding author on reasonable request.
